# Ethnomedicinal Uses, Phytochemistry, and Anticancer Potentials of African Medicinal Fruits: A Comprehensive Review

**DOI:** 10.3390/ph16081117

**Published:** 2023-08-08

**Authors:** Nosipho Thembekile Fakudze, Paromita Sarbadhikary, Blassan P. George, Heidi Abrahamse

**Affiliations:** Laser Research Centre, Faculty of Health Sciences, University of Johannesburg, Doornfontein, P.O. Box 1701, Johannesburg 2028, South Africa; 201101747@student.uj.ac.za (N.T.F.); habrahamse@uj.ac.za (H.A.)

**Keywords:** cancer, biological activities, fruits, phytochemical, ethnobotany, traditional medicine

## Abstract

Africa is home to diverse medicinal plants that have been used for generations for the treatment of several different cancers and, presently, they are gaining interest from researchers as promising approaches to cancer treatment. This review aims to provide a comprehensive review of dietary and medicinal African fruits including their traditional uses, botanical description, ethnobotanical uses, bioactive phytochemical compositions, and anticancer properties investigated to date in vitro, in vivo, and in clinical studies. Data on recent updates concerning the traditional uses and anticancer properties of these fruits were collected from a myriad of available publications in electronic databases, such as Web of Science, PubMed, ScienceDirect, Scopus, SpringerLink, and Google Scholar. The results suggest that approximately 12 native or commercially grown African fruits belonging to different plant species, including *Tribulus terrestris*, *Xanthium strumarium*, *Withania somnifera*, *Xylopia aethiopica*, *Abelmoschus esculentus*, *Carissa macrocarpa*, *Carpobrotus edulis*, *Syzygium cumini*, *Kigelia Africana*, *Annona muricata*, *Persea americana,* and *Punica granatum*, have been reported for their potential as treatment options for the management of cancer. We further found that approximately eight different fruits from native plant species from Africa, namely, *Sclerocarya birrea, Dovyalis caffra*, *Parinari curatellifolia*, *Mimusops caffra*, *Carpobrotus edulis*, *Vangueria infausta*, *Harpephyllum caffrum,* and *Carissa macrocarpa*, have been widely used for the traditional treatment of different ailments but somehow failed to gain the interest of researchers for their use in anticancer research. In this review, we show the potential use of various fruits as anticancer agents, such as *Tribulus terrestris*, *Xanthium strumarium*, *Withania somnifera*, *Xylopia aethiopica*, *Abelmoschus esculentus*, *Carissa macrocarpa*, *Carpobrotus edulis*, *Syzygium cumini*, *Kigelia Africana*, *Annona muricata*, *Persea americana,* and *Punica granatum*; unfortunately, not enough reported research data have been published to gain thorough mechanistic insights and clinical applications. Additionally, we discuss the possibility of the utilization of potential phytochemicals from fruits like *Persea americana* and *Punica granatum* in anticancer research, as well as future directions.

## 1. Introduction

Cancer is one of the leading causes of death in the world. Prostate (7.3%), breast (11.7%), lung (11.4%), skin (6.2%), and colorectal (6%) cancers are the most common types of cancer [1]. It was reported in the GLOBACON 2020 report that new cancer cases were at 19.3 million, while cancer deaths were at nearly 10 million [1]. As per the GLOBOCAN 2020 report, Africa has an incident rate of 5.7% and a mortality rate of 7.2% [1,2]. The cancer rate is predicted to rise and double by 2040, reaching approximately 1.5 million new cases [1,3]. As per the latest data for 2020, South Africa reports approximately 108,168 new cancer cases alongside 56,802 cancer-associated deaths. The most common types of cancer in the country are breast (15,491 new cases), prostate (13,152 new cases), lung (8950 new cases), colorectal (3984), and cervical (10,702) [4,5]. Breast cancer is the leading type of cancer in Black woman [4]. It has a lower incidence rate in Africa (28.66) compared to Europe (70.08) but has a higher death toll (Africa 13.72 and Europe 15.93) [6]. The 5-year survival rate for breast cancer is at 40% in low-income countries [4,5,7]. This is attributed to the frequency and access to diagnostic tools and the lack of facilities and accessibility [7,8].

Several therapies, such as chemotherapy, immunotherapy, radiotherapy, and surgical resection, have been employed in the management of cancer; however, their effectiveness has been reduced owing to drug resistance and adverse side effects [9]. Most clinically approved chemotherapeutic agents are reported to nonselectively target cells with high proliferation and regeneration abilities, such as bone marrow and skin cells, as well as epithelial cells of the digestive tract and hair, thereby triggering marked levels of toxicity linked with their treatments [10,11]. Complications, such as severe liver damage, infertility, cardiotoxicity, neurotoxicity, and nephrotoxicity, usually accompany the prolonged use of these conventional cancer treatments, which worsen the treatment outcome [12,13,14,15]. Conversely, many new potential strategies in cancer therapy have resurfaced with the ability to reduce cancer-related mortalities. A few of these strategies include gene therapy, which modifies cancer/tumor cells utilizing gene materials (e.g., plasmid DNA) or via immune cell stimulation; photodynamic therapy (PDT), which utilizes photosensitizing drugs at a specific wavelength of light to kill/eradicate cancer cells; phototherapy, where cancer cells are usually killed with the aid of heat generated by light-activated photothermal agents and nanoparticle-mediated drug therapies [16].

The use of natural products such as plants has been around since prehistoric times, as early as 60,000 years. Medical systems, such as traditional Chinese medicine, have utilized formulations from natural products for as long as thousand years (5000) and have refined the dosages, preparation, and processing [17]. Secondary metabolites discovered through biochemical analysis of plant extracts and products using cutting-edge methods (mass spectrometry analysis) can be potential agents for the successful treatment of several diseases. These phytochemicals can include phenols, flavonoids, steroids, and more [18]. These secondary metabolites have been studied and shown to have antitumor, antiproliferation, and preventative effects on numerous cancers [19,20,21,22]. According to certain reports, it is estimated that 60% of the world’s population uses traditional medicine, while 80% of people still rely on plant-based products for their primary healthcare. Africa has always been regarded as the treasure trove of medicinal plants, with over 5400 known and documented medicinal plants [23]. A large population in Africa frequently uses medicinal plants for cancer treatment because of a low income or long distances from urban treatment centers, as well as the history of African cultures and beliefs that medicinal plants are superior to pharmaceuticals in treating diseases like cancer. In traditional medicine, patients are treated differently in various parts of Africa, and this largely depends on the plants, protocols, and recipes that are employed, whereby the most frequently used explants are the roots and stem barks [24]. Surprisingly, fruits have been explored to a much lesser extent than all other plant parts. Thus, in this review, we aimed to discuss some of the fruits that are either indigenous or cultivated majorly in Southern Africa or other parts of Africa which have been studied for their anticancer properties or have the potential for further investigation. Additionally, we summarized research that identified the mechanisms by which different phytocomponents of fruits exert their anticancer properties. The search criteria for the literature was limited to the years 2008–2023 in electronic databases such as the Web of Science, PubMed, ScienceDirect, Scopus, SpringerLink, and Google Scholar. Search words included terms such as “anticancer”, “phytochemicals”, “traditional uses”, and “indigenous African fruits”. Plants/fruit that were not cultivated or indigenous were excluded from search criteria. Our initial search results included native Southern African plant species, such as *Sclerocarya birrea, Dovyalis caffra*, *Parinari curatellifolia*, *Mimusops caffra*, *Carpobrotus edulis*, *Vangueria infausta*, *Harpephyllum caffrum*, and *Carissa macrocarpa,* whose fruits have been used in general traditional medicine for generations. However, an in-depth literature search showed a lack of studies on these fruits with respect to anticancer efficacy. As a result, we expanded the scope of our search to include both native and widely cultivated plant species in Africa, whose fruits have been investigated for their anticancer potency.

## 2. Fruits in Cancer Treatment

Cancer is a burden in South Africa and as part of the United Nations Sustainable Development Goals has been made a priority [5]. A strategic framework has been added nationally to address this burden [5]. Radiotherapy, surgery, chemotherapy, targeted therapy, and hormonal therapy are the available treatment methods but still negated because of the diagnosis of cancer in an advanced stage, cost of treatment, poor treatment access, and lack of treatment completion [4,5,25,26]. Natural phytoproducts have a long history of being used for both preventative and therapeutic purposes in medicine and are recognized as the cornerstone of both conventional medical treatments and the primary building blocks of lead compounds in drug discovery [27]. Because of the nature of phytochemicals having antitumor effects, they have been made popular in the medical field for prevention and curative compounds against cancer, and a few have been given approval for clinical use [18,28,29,30,31]. Their ability to control numerous survival pathways with fewer negative effects is an essential component of their function. Four major plant-derived antitumor agents are vinca alkaloids, camptothecin derivatives, epipodophyllotoxin, and taxanes diterpenoids [18,32]. Biological macromolecules (approximately 21%), unaltered natural products (7.3%), natural product botanical (0.4%), and natural product derivatives (17.4%) are part of the 247 approved anticancer drugs as of 2019.These show possibilities of utilization of phytochemicals with antitumor properties in future cancer treatment research [33].

South Africa is a country that practices both Western and traditional medicine. Traditional medicine uses medicinal preparations of indigenous plants, vegetables, and fruits [34]. This is made easy by the abundance of such vegetation in the country [34,35]. These plants/fruits have not all been explored for their medicinal properties, and there is the prospect of finding a remedy that is easily accessible and plentiful [34,36]. These remedies are utilized to treat ailments such as fever, sinusitis, indigestion, asthma, diarrhea, and skin diseases [37]. Successful treatment of diseases using plants/fruits is linked to the phytochemicals they contain. Fruits contain phytochemicals like flavonoids, saponins, anthocyanins, terpenoids, phenolic acids, and porphyrins [37,38]. Phytochemicals are important to the medical field because of their healing properties and have recently been of focus for the treatment of numerous ailments from infections to cancer [34,36,38].

Several fruits that are consumed for medicinal or dietary purposes are a great source of bioactive phytocompounds like anthocyanins, ellagitannins, flavonols, proanthocyanidins, and phenolic acids. These phytochemicals have both prevention of tumorigenesis and cancer treatment properties exerted by several different mechanisms, including inhibiting carcinogen-induced DNA damage, defense against oxidative DNA damage, and modulation of signaling pathways involved in cellular proliferation, inflammation, angiogenesis, cell cycle arrest and death, regulation of xenobiotic-metabolizing enzymes, various transcription and growth factors, inflammatory cytokines, and other subcellular signaling pathways [39]. In Table 1, we summarize the list of fruit plants native to or primarily grown in Southern Africa or other regions of Africa and their traditional use in folklore medicine.

### 2.1. Biological Activities of Southern African Fruits

#### 2.1.1. Marula

The marula fruit belongs to *Sclerocarya birrea* (A. Rich) Hochst, subspecies *caffra*. This tree grows in sub-Saharan Africa and can be found in Limpopo, Kwa-Zulu-Natal, Eastern Cape, and Mpumalanga in South Africa. This plant is a medium-sized to bulky tree that typically reaches 9 to 18 m in height [40]. The fruit turns from green to waxy yellow when ripe during January to March. The fruit is plum sized, with a thick peel and white, sweet and sour, pulpy inner flesh [41]. The phytochemicals contained in marula are tannins (procyanidin dimer B1 and procyanidin B5), xanthohumol A, phenolic acids, anthocyanin derivatives flavonoid aglycones (catechin and epicatechin), flavonoid glycosides (myricetin 3-arabinoside, quercetin 3-galactoside, quercetin-3-O-rutinoside, and myricetin 3-galactoside) [42]. Traditionally goiter, diarrhea, hypertension, indigestion, ulcers, dysentery, anemia, proctitis, scurvy, diabetes mellitus, malaria, fever, and fungal infections are treated with decoctions from marula [37,40]. Marula has antioxidant, antibacterial, antifungal, and antibacterial properties that are responsible for the curative effects that lead to its use in traditional medicine [43,44,45]. *Sclerocarya birrea* (A. Rich) Hochst bark extract had a minimum inhibitory concentration (MIC) of 188 mg/mL against *Staphylococcus aureus*, 200 mg/mL against *Pseudomonas aeruginosa*, 140 mg/mL against *Enterococcus faecalis*, and 39 mg/mL against *Escherichia coli,* showing significant success in its potential use as an antibacterial product/medicine [46].

The toxicity of *S. birrea* extracts has been the subject of several studies. Aqueous and methanolic extracts of *S. birrea* stem–bark administered intraperitoneally to mice resulted in LD_50_ values of 1215 mg/kg and 1087 mg/kg, respectively, indicating its relatively “safe” status [47]. However, oral administration of doses of 3000 and 4000 mg/kg body weight of the fruit peel extract in distilled water showed toxic effects on the liver and kidneys in rats [48]. Hexane, methanol, and water extracts of the plant’s stem–bark (10–1000 mg/mL) did not show any toxic effects against brine shrimps in a brine shrimp lethality assay [49]. The LD_50_ values for *S. birrea* stem–bark methanol extracts were above 5000 mg/kg body weight upon oral administration of doses in mice [50]. Another study showed that prolonged exposure to high doses of stem–bark ethanolic extract (600–1000 μg/mL) in kidney cell lines of the proximal (LLC-PK1) and distal tubules (MDBK) reduced cell viability. However, the same extract at a concentration of 120 mg/kg had no discernible impact on renal fluid and electrolyte handling in nondiabetic and STZ-treated diabetic rat models. This was explained as excretion of weak acidic and phenolic substances in an in vivo model, while in vitro cells were continuously exposed to phenolic substances [51]. Further, a 3-week supplementation regimen of *S. birrea* fruit juice showed significant reductions in total serum cholesterol (8%), LDL cholesterol (17%), and triglycerides (7%), as well as an increase in HDL cholesterol concentration (by 10%) and a reduction in serum oxidative stress in healthy individuals. The antioxidant fractions were shown to contain high amounts of hydrolysable tannins, catechins, and derivatives of hydroxycinnamic acid, which may be responsible for its antioxidant efficacy [52].

#### 2.1.2. Kei Apple

*Dovyalis caffra* (Hook.f. & Harv.) Hook.f., commonly identified as kei apple, belongs to the Flacourtiaceae family [53]. The fruit can be found in the Limpopo, Mpumalanga, Eastern Cape, and Kwazulu Natal provinces of South Africa [41]. *D. caffra* is a dense, thorny shrub that averages 3–5 m tall but can occasionally grow up to 8 m. Its flowers have no petals and are small. The fruit peel is rough, smooth, and bright yellow. The fruit is round and up to 4 cm in diameter with juicy, apricot-like flesh, which ripens from December to January [37,54]. Its phytochemical profile includes phenolic acids (F2) (anthocyanin monomers, procyanidins, and catechins), anthocyanin polymers, and flavonols [53,55]. It is traditionally utilized in the treatment of chest pain, amenorrhea, rheumatism, and pain-associated issues [56]. Studies show kei apple has potential as an antiviral, antioxidant, antimalignant, and antimicrobial agent [57]. *D. caffra* methanol fruit extract is rich in chlorogenic acid (2107.96 ± 0.07 µg/g), catechin (168 ± 0.58 µg/g), and gallic acid (15.66 ± 0.02 µg/g). Its major bioactive phytocompounds were tested for their antimicrobial properties via the disc diffusion method, whereby the dried methanolic extract loaded at a dose of 20 µg/disc showed inhibition zones of 15 mm, 13 mm, 22 mm, and 24 mm for *Staphylococcus aureus*, Bacillus *subtilis*, *Escherichia coli,* and *Proteus vulgaris*, respectively. Additionally, its antioxidant activity was indicated with 2,2-diphenyl-1-picryl-hydrazyl-hydrate (DPPH) scavenging at 79.25% and an inhibitory concentration 50 (IC_50_) of 728.20 ± 1.04 g/mL at a concentration of 2000 μg/mL and 1000 µg/mL, reaching a 41.10% viability. Treatment of HepG2 cancer cells at 1000 μg/mL showed potential anticancer activity of the extract with a ~58.90% loss in cell viability [57]. Leaves and branches extracts of this plant exhibited no cytotoxicity against HepG2 cells at the final concentration of 100 μg/mL [58], while another study showed antiproliferative activity of a methanolic branch extract with an LC_50_ of 37 μg/mL, 72 μg/mL, 43 μg/mL, and 20 μg/mL against MCF-7, HCT-116, HepG2, and A-549, respectively [59]. A study showing the antioxidant effect of *D. caffra* fruit extract against free radicals showed that the whole fruit extract’s DPPH scavenging action peaked at 52.11% (IC_50_ 95.09 μg/mL) at a concentration of 100 μg/mL, while the flesh extract’s action was 27.11% (IC_50_ 187.12 μg/mL) at the same concentration. The two main phenolic compounds in dry fruits that were identified were chlorogenic, pyrogallol, and malic acids as the most prevalent organic acids. Further, the results of the acute toxicity demonstrated that the water:methanolic extract of whole Kei-apple fruit is safe, with an LD_50_ value of more than 6000 mg/kg body weight when administered orally to male and female rats. [60].

#### 2.1.3. Mobola Plum

*Parinari curatellifolia* Planch. ex Benth, generally known as mobola plum, is part of the Chrysobalancaceae family. The fruit can be found in Pondoland in Kwazulu Natal, South Africa [61]. It is a tree/shrub that can range from 3 to 20 m. The tree/shrub is an evergreen plant, with a small white flower with a touch pink. Its tree has a sporadic pungent smell. When ripe, the drupe-shaped fruit is yellow–orange with grey specks. The scaly-textured, oval or round fruit, which can take up to a year to ripen, is found at the ends of twigs. These roughly 50 mm long plum-like fruits have yellow edible flesh. When fully ripe, the fruits have a pleasant flavor, and they typically ripen on the ground from October to January [54,62,63]. This plant/fruit has been traditionally used and studied for its anti-inflammatory, antimicrobial, antioxidant, and disease preventive effects, such as reduction in cardiovascular risk and in the treatment of fever, diabetes mellitus, hypertension, body aches, and piles [62,64]. Mobola plum consists of phytochemicals such as flavonoids, coumarins, polyphenols, tannins, glycosides, saponin, alkaloids, anthraquinones, and terpenoids [65,66]. *P. curatellifolia* fruit methanol extract showed the strongest antioxidant properties, as represented by 9.5 TEAC/g and 14.2 IC_50_ µg/mL, determined from ferric-reducing ability of plasma (FRAP) and DPPH scavenging activity assays. The strong antioxidant activity of the extract is associated with a high content of phenolics identified as neochlorogenic acid, chlorogenic acid, 3-O-p-coumaroylquinic acid, 3-O-caffeoylshikimic acid, delphinidin 3-galactoside, delphinidin 3-O-glucoside, cyanidin 3,5-O-diglucoside, catechin, epicatechin, myricetin 3-galactoside, quercetin 3-galactoside, quercetin 3-O-α-l-arabinopyranoside, gentisic acid 5-O-glucoside, procyanidin dimer B1, procyanidin B5, procyanidin B-type dimer, β-glucogallin (1-O-galloyl-β-d-glucopyranose), and tryptophan [67]. The inhibitory effect of angiotensin-I-converting enzyme by *P. curatellifolia* seed crude extract at IC_50_ 13.54 μg/mL showed positive antihypertensive properties [64]. The cytotoxicity study demonstrated the antiproliferative effect of the aerial plant part with methanol extract (IC_50_ 48.5 μg/mL) and petroleum ether extract (1.503 µg/mL) against HeLa cells [68]. An evaluation of the acute toxicity and subchronic toxicity of a single dose of *P. curatellifolia* seed hydroethanolic extract in mice showed an LD_50_ of 7.27 g/kg body weight, indicating its potential safety. However, the study further showed the potential for kidney damage at higher doses, resulting in renal failure and toxic effects on the heart during long-term treatment [69]. The LD_50_ values of the methanol and ethyl acetate extracts administered via the intraperitoneal route in mice were found to be 113 mg/kg and 471.17 mg/kg, respectively [70]. Oral acute administration of the methanolic stem–bark extract of *P. curatellifolia* revealed an LD_50_ dose > 5000 mg/kg in rats; therefore, it is considered to generally be safe. However subchronic administration of the extract showed long-term use may harm the liver, which was revealed by mild infiltrating leukocytes, vascular congestion, and piece meal necrosis [71].

#### 2.1.4. Red Milkwood

Red milkwood in South Africa can be differentiated into two types: *Mimusops caffra* E. Mey. ex. A.DC. and *Mimusops zeyheri* Sond. Both of these species belong to the Sapotaceae family. They are also known as the coastal and the Transvaal red milkwood. *M. caffra* is found in coastal KwaZulu-Natal in South Africa, while *M. zeyheri* bushveld and woodland is found in the northern part of South Africa [72,73]. The evergreen tree is grown in large uniform clusters. The tree can be small to medium in size and can grow up to 10–15 m [72]. The fruit is characterized by one-seeded, 2.5 cm × 1.5 cm, and ovoid-shaped berries with an orange-to-red coloration when ripe, and they are available between September and December. The ripe fruits are palatable and delightfully sweet, and they are consumed by humans, monkeys, and many bird species [72,74,75]. Phytochemicals found in red milkwood include saponins, flavonoids, and polyphenolics [76]. The plant has been reported to have anesthetic, antifungal, and protective properties, such as gastric, in folklore medicine [76]. Red milkwood is traditionally used to treat wounds, sexually transmitted infections, tuberculosis, uterine issues, fungal infections (candidiasis), and malaria, as well as used as an emetic [74,76,77,78]. Studies on the plant’s biological activities have not been well documented [76].

#### 2.1.5. Sour Fig

Sour fig (*Carpobrotus edulis* (L.) L. Bolus) belongs to the Aizoaceae family. *C. edulis* is native to South Africa but has been transplanted to different subtropical countries to control erosion [79]. Along the coastline of the Cape (eastern, western, northern, and south) of South Africa [80], the plant bears yellow flowers that turn into a reddish brown (ripe) pointed encapsulated fruit in the spring and summer (August to October). The fruit measures 35 mm in diameter, is indehiscent and edible, and is shaped like a spinning top. When ripe, the fruit turns yellow and becomes fragrant. With time, the fruit’s exterior wall turns leathery, wrinkly, and yellowish. The seeds are enmeshed in the mucilage, which is gummy, sweet, and jelly-like. The fruits have a strong, astringent, salty, and sour taste and can be consumed fresh [79,81]. Phytochemicals isolated from the leaves, stems, and flowers of sour figs include isoterpinolene, naphthalene, 1,2-dihydro-2,5,8-tri, bistrimethylsilyl N-acetyl EICOSAS, β-amyrin, oleanolic acid, uvaol, monogalactosyldiacylglycerol, (acyls = linolenoyl group; MGDG), epicatechin, N-octanol, nonylaldehyde, trans-β-demascenone, trans-2-tridecenal, tetradecamethylcycloheptasiloxane, tetradecamethylcycloheptasiloxane, tetradecamethylcycloheptasiloxane, octadecane, octadecane, 1-octadecene, nonadecane, 2-pentadecanone,6,10,14-trimethyl, eicosane, eicosane, phytol (2-hexadecen-1-o1,3,7,11,15-tetramethyl), tetrasiloxane,1,1,1,5,7,7,7-heptamethyl-3bis(trimethylsilyl)oxy, tetracosamethylcyclododecasiloxane, phenolics (procyanidins, flavonoids sinapic acid, hyperoside, ferulic acid, isoquercitrin, and ellagic acid), hydrolysable tannins, alkaloids, and saponins [80,82,83]. *C. edulis* has been used in traditional medicine to treat tuberculosis, dysentery, stomach aches, throat infections, burns, mouth ulcers, and sinusitis [82,83,84]. *C. edulis* is a plant that has been documented to have antifungal, antioxidant, antiplasmodial, anticancer, and antibacterial properties [83]. A hexane, acetone, ethanol, and water extract of C. edulis leaf tested as an antifungal agent showed higher concentrations of ethanol extract (1.25 mg/mL) and inhibited the growth of *Candida krusei* and *Candida albicans* [85]. An aqueous extract of *C. edulis* leaf exhibited the highest antibacterial activity against *Staphylococcus aureus* (MIC 0.4%) followed by *Escherichia coli* (MIC 1.6%) and *Pseudomonas aeruginosa* (MIC 6.6%) at a concentration of 600 mg. Six isolated compounds from *C. edulis*, including uvaol, β-amyrin, oleanolic acid, catechin, epicatechin, and monogalactosyldiacylglycerol, were extremely active against specific Gram-positive bacteria and had moderate activity against *Mycobacterium tuberculosis*, while there was no activity against Gram-negative bacteria. High antibacterial activity was shown by oleanolic acid against a variety of bacterial strains like *Enterococcus faecalis* (MIC 6.25 mg/L), methicillin-resistant *Staphylococcus aureus* MRSA COL (MIC 50 mg/L), MRSA COL resistance to oxacillin MRSA COL_OXA_ (MIC 25 mg/L), *S. aureus* HPV 107 (MIC 25 mg/L), and *M. tuberculosis* H37Rv (MIC 100 mg/L). Interestingly, uvaol showed the greatest ability to reduce the multidrug resistance of the MRSA COL_OXA_ strain because of its effect on the efflux pump system [84]. Hexane, acetone, ethanol, and distilled water extracts of *C. edulis* leaf demonstrated strong antioxidant activity in the concentration range of 0.025–0.5 mg/mL, determined with FRAP, DPPH, and 2,2-azino-bis-3-ethylbenzothiazoline-6-sulphonic acid (ABTS) assays [82]. The antiproliferative activity of *C. edulis* leaf aqueous extract inhibited the cell viability of neuroblastoma cell lines SK-N-BE(2) at an IC_50_ 0.86 mg/mL and SHSY5Y at an IC_50_ 1.45 mg/mL through the induction of mitochondrial-mediated apoptosis and the accumulation of intracellular ROS [86]. Further, a toxicity study with a methanol extract of *C. edulis* leaves with different dilutions showed no toxicity till 3 days of treatment and was evident only after day 5 in the THP-1 cell line and human peripheral blood monocyte-derived macrophages [87].

#### 2.1.6. Wild Medlar

*Vangueria infausta* Burch. subsp. *infausta* belongs to the Rubiaceae family and is commonly known as wild medlar. It grows/is found in the KwaZulu-Natal, Eastern Cape, North-West, Limpopo, Free State, and Gauteng provinces of South Africa. Wild medlar is a small tree/shrub that reaches up to 3–8 m height. Its flowers are a green-tinted white to yellow [88]. The tree bears pendulous (downward hanging) clusters of nearly spherical green fruits that turn yellow–brown when ripe from January to April, and they can reach a diameter of 25 mm. [89,90]. Wild medlar has phytochemicals such as tannins (procyanidin B5, procyanidin dimer B1, procyanidin B-type dimer), xanthohumol A, terpenoids, coumaroylquinic acid, caffeoylshikimic acid esters, saponins, anthocyanin derivatives, flavonoid aglycones (catechin); flavonoid glycosides (quercetin-3-O-rutinoside), aromatic amino acid tryptophan, and phenolic acids extracted from fruits, root bark, aerial shoots, leaves, and roots. [67,88,89,91]. Wild medlar has been utilized to treat roundworm, snake bites, malaria, fever, fungal infections (candidiasis), pneumonia, and chest-related problems in traditional medicine [56,88,92]. The successful use of wild medlar in traditional medicine can be traced back to its antioxidant, antibacterial, antifungal, antiplasmodial, and anti-inflammatory properties [88,93,94,95,96,97,98,99,100]. The cytotoxicity activities of methanolic and aqueous root extracts of this plant against MAGI CC5+ cells showed cytotoxic half-maximal concentrations (CC50) of 0.1 mg/mL [101]. Dichloromethane and methanol extracts of roots of *V. infausta* tested against Rat skeletal myoblast L6 cell line showed IC_50_ values of 45.7 μg/mL and 71.5 μg/mL, respectively [102]. Acetone extract of the dried leaves showed half maximal lethal concentration (LC_50_) values of 0.074 mg/mL and 0.763 mg/mL for Vero kidney cells and C3A liver cells, respectively [103]. Ethanol leaf extracts demonstrated an LC_50_ value of 144.7 μg/mL in a lethality test using brine shrimp, which was considered to be nontoxic compared to the cyclophosphamide LC_50_ value of 16.3 μg/mL [104]. According to these early cytotoxicity assessments conducted thus far, extracts of *V. infausta* is characterized by low levels of cytotoxicity.

#### 2.1.7. Wild Plum

*Harpephyllum caffrum* Bernh. of the Anacardiaceae family is generally called wild plum [105]. Wild plum grows in the KwaZulu-Natal, Limpopo Eastern, and Cape parts of South Africa [106,107]. *H. caffrum* is an evergreen tree of short to medium size that reaches 6 to 15 m [54]. It has small, white to yellow–green flowers [54]. Its fruit is single-seeded, oblong (25 × 13 mm), thin, fleshy, and plum-like drupe that first appear green and then turn red when they ripen in March–August [54,108]. Its phytochemical profile includes phenolic acids, anthocyanins, flavonoids, hydrolysable tannins, and glycosides [38]. This plant has been used traditionally to treat acne, epilepsy, eczema, and childhood tremors, as well as a pain blocker and for sores [37]. Wild plum has been researched and shown to exhibit anti-inflammatory, anesthetic, antimicrobial, and antifungal activities [109]. A study conducted by Nawwar and colleagues showed that the antioxidant effect of the aqueous methanol leaf extract of *H. caffrum* showed protection of the human keratinocyte cell line, HaCaT, against UV phototoxic response at 40 μg/mL, while negligible cytotoxicity was observed at the highest concentration of 100 μg/mL [105]. In one study, oral administration of the leaf ethanol extract of *H. caffrum* did not result in any mortality in rats up to 10 g/kg body weight and was, therefore, deemed safe. Further, this extract demonstrated hepatoprotective activities at a daily dose of 100 mg/kg body weight. This extract also showed antiproliferative activity against HEPG2 (IC_50_ 1.21 μg/mL), HEP2 (IC_50_ 1.34 μg/mL), and HCT 116 (IC_50_ 3.62 μg/mL) [109]. The ethanolic extract of *H. caffrum* leaf showed ~80% toxicity at 100 μg/mL, while no toxicity was observed with bark extract when tested against B16-F10 melanocyte cells [110].

#### 2.1.8. Natal Plum (Num Num)

The natal plum, or num-num (*Carissa macrocarpa* (Eckl.) A.DC.), belongs to the Apocynaceae family [111]. *C. macrocarpa* grows in Southern Africa and can be found in Mpumalanga, Free State, KwaZulu-Natal, Gauteng, North West, Western Cape, Limpopo, and Eastern Cape provinces of South Africa [112]. This short evergreen shrub grows up to 5 m with pale yellow flowers that produce large, oval, and red plum-like fruits in summer [111,112]. Its phytochemical profile consists of steroids, alkaloids, saponins, flavonoids, carbohydrates, tannins, phenols, triterpenoids, and quinones [113,114]. *C. edulis* is typically used to treat issues such as an analgesic, malaria, dysentery, stomach ache, coughing, cataracts, sexually transmitted infections, and cancer [115]. The literature suggests that, compared to other species of *Carissa,* biological studies on *C. macrocarpa* has not been well documented [113,116,117]. Oleanane triterpenes and ursolic acid isolated from fruits and leaves extracts of *C. macrocarpa* showed good antibacterial activity against bacterial strains of *Klebsiella pneumoniae*, Pseudomonas aeruginosa, *E. coli*, *Staphylococcus saprophyticus*, *Staphylococcus aureus*, and *Enterococcus faecium* at MICs ranging from 0.06 mg/mL to 1.0 mg/mL [118]. The hydroethanolic extract from *C. macrocarpa* fruits inhibited the growth of the tumor cell lines NCI-H460 (GI_50_ 57 μg/mL), HeLa (GI_50_ 66 μg/mL), MCF-7 (GI_50_ 109 μg/mL), and HepG2 (GI_50_ > 400 μg/mL) without inducing toxicity in nontumor porcine liver cells (PLP2) [119]. The cytotoxic activity of hydroethanolic extracts of the leaves, stems, and flowers of *C. macrocarpa* showed 50% cell growth inhibition dose (GI_50_) values ranging from 52.1 to 167 μg/mL for the four tested tumor cell lines MCF-7, NCI-H460, HeLa, and HepG2 [113].

**Table 1:** Summary of indigenous fruits and their distribution in Africa.

Name	Indigenous Name (South Africa)	Distribution in Africa	Plants Parts Used for Treatment of Disease Conditions in Folk Medicine	References
*Sclerocarya birrea*	Marula, umganu (in Zulu)	South Africa, Botswana, Zimbabwe, Namibia, Zambia, Eswatini, Malawi, and Mozambique	(1) Bark: to treat diabetes, snake bite, pruritis, pharyngitis, splenomegaly, goiter, cholera, dysentery, diarrhea, proctitis, stomach ailments, ulcers, inflammation, arthritis, hypertension, skin diseases, fever, malaria, and proctitis; (2) roots: to treat snake bite, pruritis, pharyngitis, splenomegaly, goiter, fungal infections, and sore eye; (3) leaves: cold and flu, snake bite, pruritis, pharyngitis, splenomegaly, goiter, diarrhea, dysentery, proctitis, stomach ailments, ulcers, inflammation, arthritis, hypertension, skin diseases, fever, malaria, diabetes, fungal infections, and heartburn; (4) fruit: snake bite, pruritis, pharyngitis, splenomegaly, and goiter.	[40,43,44,56]
*Dovyalis caffra*	Kei Apple, umqokolo (in Zulu)	Eswatini, Malawi, South Africa, Zimbabwe, Mozambique, Tanzania, Lesotho, and Namibia	(1) Bark: to treat rheumatism; (2) root: to treat amenorrhea, chest pain, and rheumatism; (3) fruit: to treat coughing in different livestock; (4) thorns: amenorrhea and chest pain.	[55,56]
*Parinari curatellifolia*	Mobola Plum, mmola (in Sotho)	Zimbabwe, South Africa, Malawi, Nigeria, and Eswatini	(1) Bark: to treat pneumonia, cataracts, and earache; (2) leaves: to treat dislocated bones, broken bones, wound, and pneumonia.	[64,65,67]
*Mimusops caffra*	Red Milkwood, umThunzi (in Zulu)	Botswana, Mozambique, Tanzania, Eswatini, Angola, Zimbabwe, and South Africa	(1) Bark: to treat wounds and sores, and used as an emetic; (2) root: to treat sexually transmitted infections, such as gonorrhea, and other ailments, like candidiasis, tuberculosis, weight loss, and womb problems; (3) Leaf: antiplasmodial activity and used to manage malaria.	[74,75]
*Carpobrotus edulis*	Sour Fig, igcukuma (in Xhosa)	Coastal South Africa to coastal Mozambique	Leaves: to treat diarrhea, stomach upset, tuberculosis, skin infections (eczema, dermatitis, and sunburns), sinusitis, vaginal thrust burns, and toothache.	[37,81,82,83]
*Vangueria infausta*	Wild Medlar, umtulwa (in Zulu)	Malawi, Uganda, Tanzania, Mozambique, Kenya, Zimbabwe, Botswana, South Africa, Lesotho, Eswatini, and Namibia	(1) Roots: to treat abdominal pains, asthma, blood pressure, chest pain, cold and cough, diabetes, diarrhea and stomach problems, epilepsy, fever, headache, hernia, infertility, malaria, measles, male virility, menstrual problems, nervous system disorders, candidiasis, pneumonia, skin blisters, snake bites, stomach ulcers, and parasitic worms; (2) bark: skin blisters, syphilis, asthma, bloody stool, blood pressure, chest pain, cold and cough, diarrhea and stomach problems, infertility, and candidiasis; (3) leaf: to treat abscesses, asthma, blood pressure, chest pain, cold and cough, dermatitis, diabetes, fever, headache, hernia, malaria, candidiasis, parasitic worms, pleurisy, pneumonia, skin blisters, swellings, and toothache; (4) fruit: to treat menstrual problems and parasitic worms.	[56,88]
*Harpephyllum caffrum*	Wild Plum, umgwenya (in Zulu)	South Africa, Eswatini, Mozambique, and Zimbabwe	(1) Bark: to treat acne and eczema, sprains, and bone fractures; (2) root: to treat paralysis caused by sorcery.	[107,120]
*Carissa macrocarpa*	Num Num or Amatungula in IsiZulu	South Africa and Mozambique	Leaves: to treat venereal diseases and coughs.	[121,122]

## 3. Anticancer Effects and Cellular Targets of Selected African Fruits

Unfortunately, because of several unknown reasons, despite the wide traditional use of these fruits and/or their extracts, they have not been explored for their anticancer properties. Therefore, because of a lack of available information, in this section we have tried to compile and provide a list of Southern African, as well as other African, fruits studied for their anticancer potential (Table 2).

### 3.1. Tribulus terrestris *L*.

*T. terrestris* (puncture vine) is a perennial herb that belongs to the Zygophyllaceae family and is indigenous to tropical regions, including Africa and South Africa [123,124]. It is a short, prostrate shrub with silky hair that grows 10 to 60 cm tall. Its greenish yellow carpel fruits have a distinctive, stellate shape, are somewhat rounded and compressed, have five corners, and are covered in very pale yellow prickles. Each crocus has several seeds with transverse partitions separating them [125]. The fruits have been used to treat oxidative stress; kidney, liver, and cardiovascular diseases; skin pruritus; headache and vertigo; and mammary duct blockage, infertility, impotence, erectile dysfunction, low libido, nephritis, and inflammatory disorders. The plant parts have also been used as an antiaging agent, a diuretic and cough expectorant, and an aphrodisiac and hormone booster in both men and women [124,126].

Several extracts of this plant, because of their high content of steroidal saponins, have been shown to induce its antineoplastic effects in both in vitro and in vivo studies against a wide range of human cancer cells [24,127,128]. Furthermore, fruit extracts of *T. terrestris* extracted in acetone and methanol have also shown to negatively affect the proliferation, growth, migration, and invasion of cancer cells by targeting the cellular autophagy protease ATG4B (autophagin-1) to block autophagy pathway [129,130]. A study conducted by Kim et al. showed that aqueous fruit extract of *T. terrestris* induces G0/G1 cell cycle arrest and apoptotic cell death resulting in a strong antiproliferative effect on HepG2 cancer cells. Mechanistic experiments revealed the involvement of extract-mediated regulation of certain signaling pathways: (1) degradation of cyclin E/cdk2 and cyclin D/cdk4 complexes resulting in cell cycle arrest; (2) changing the Bax/Bcl-2 ratio activating the apoptotic cell death pathway; (3) inhibition of the MMP-2 and MMP-9 expression blocking tumor invasion and metastasis; and (4) suppressing the activity of NF-κB by decreasing the level of p50 and causing inhibition of the NF-κB-regulated expression of genes involved in cell proliferation, anti-apoptosis, and invasion [131]. A carcinogen-induced skin papillomagenesis model using Swiss albino mice has also been used to demonstrate the tumor chemopreventive potential of *T. terrestris* aqueous fruit extract. According to the study, oral administration of the extract at a concentration of 800 mg/kg body weight led to a significant decrease in the number of papillomas and tumor burden while also significantly lengthening the average latent period. As suggested, the underlying mechanism was put forth as a result of altered antioxidant levels, which included a significant increase in the reduced glutathione content and a decrease in the levels of lipid peroxidation [132]. Consumption of this fruit shows adverse effects on the lungs [133].

Several toxicological studies have been carried out in vitro and in preclinical animal models [124,134]. An acute toxicity study with a methanol extract of the aerial part of the plant administrated orally at 2 g/kg of body weight of rabbits for 14 days showed no toxic symptoms or mortality [135]. Saponin-rich butanol extracts of *T. terrestris* fruit showed no acute toxicity up to 2000 mg/kg in rats. Further, the same extract given orally for 28 days at doses of 100, 200, and 400 mg/kg/day showed no adverse effects in animals. This suggests the nontoxic nature of these extracts [136]. Treatment of normal and diabetic rats with 50 mg/kg hydroalcoholic extract of *T. terrestris* for 8 weeks showed nephrotoxic effects due to the unhydrolyzed fraction of saponins in the plasma and kidney [137]. In another study conducted in sheep fed with 80% *T. terrestris* and 20% alfalfa hay and wheat straw, they developed hepatogenous photosensitivity. The symptoms included conjunctivitis, depression, jaundice, weight loss, and reddening of the muzzle, nose, ears, and eyelids. Further, the necropsy of the experimental animals showed that the livers were enlarged and stained with bile pigment to varying degrees of generalized icterus [138]. The potential cytotoxic, genotoxic, and endocrine-disrupting properties of *T. terrestris* in vitro were studied by Abudayyak et al. The results showed that compared to water and chloroform extracts, the whole plant and methanol extract induced relatively higher genotoxic (2400 μg/mL) and cytotoxic (IC_50_ = 160 μg/mL) activities but did not impair DNA in NRK-52E cells, whereas 300 μg/mL of the water extract could cause frame shift mutations when metabolically activated. Further, the extract demonstrated estrogenic activity at concentrations greater than 27 μg/m. These results suggest the toxic potential of *T. terrestris* [139].

A case study reported *T. terrestris*-related liver, kidney, and nervous system damage in a patient who used the plant’s extract to prevent kidney stones. Consumption of *T. terrestris* water for two days caused seizures, extremely high serum aminotransferases, and high levels of creatinine. When the herbal remedy was stopped, his symptoms improved, and his liver enzyme levels returned to normal [140]. A 36-year-old man who took a herbal supplement based on a *T. terrestris* extract was diagnosed with a 72 h priapism in a published case presentation [141]. Another report also presented a case of *T. terrestris* induced toxicity in a young, healthy male who developed severe hyperbilirubinemia, acute renal failure, and casts containing bile in the tubules [142]. These results suggest to raise public awareness of the potential dangers of non-FDA-approved *T. terrestris*-containing herbal supplements.

### 3.2. Xanthium strumarium *L*.

*X. strumarium* (bur weed), which belongs to the Asteraceae family, is distributed worldwide, most commonly in temperate zones, while it is considered to be invasive in parts of Africa like Kenya, Uganda, and Tanzania. However, this species has been listed as a noxious weed in South Africa [143]. It is an annual herb that can grow up to 1 m tall and has a short, stout, and hairy stem and grows typically in warmer climates. The fruits have two hooked beaks and hooked bristles and are obovoid in shape, enclosed in a hardened involucre. The anodyne, antirheumatic, antisyphilitic, appetizing, diaphoretic, diuretic, emollient, laxative, and sedative properties of the leaves and roots are used traditionally. The main medicinal component of *X. strumarium* is its fruit, which has been used for thousands of years to treat several different ailments traditionally including headaches, diseased kidneys, tuberculosis, allergic rhinitis, sinusitis, urticaria, catarrh, rheumatism, rheumatoid arthritis, constipation, diarrhea, lumbago, leprosy, eye diseases, and pruritis [144,145].

Chloroform and methanol extracts of fruits from *X. strumarium* showed strong antiproliferative and antimetastatic properties by inhibiting ATG4B proteolytic activity and autophagic flux [129,130]. Fruit extracts of *X. strumarium* are rich in sesquiterpenes, lignanoids, glycosides, thiazides, phenolics, etc. [144]. Furthermore, evaluation of methanol crude extract and ethyl acetate fraction of methanol extract of *X. strumarium* seeds on different cancer cell lines showed cell proliferation inhibition in a dose-dependent manner, whereby the ethyl acetate fraction was reported to be relatively more effective. The inhibitory effect can be due to the presence of 3,4-dihydroxybenzaldehyde, as reported in another study [146]. In a study conducted on eight pentacyclic triterpenoids isolated from the fruits of *X. strumarium*, they demonstrated strong in vitro cytotoxic activity on different cancer cells either resulting in cell cycle arrest in the G1 phase and/or induced cell death via the apoptotic pathway [147]. Karagoz et al. extensively investigated the antitumoral effects of dichloromethane and acetone extracts of *X. strumarium* fruits and pure secondary metabolites, such as xanthanolides (xanthinosin, xanthatin, xanthinin, and xanthinol) and triterpene (stigmasterol) isolated from the acetone extract of fruits. Cytotoxicity studies conducted on C6 rat glioma cells and HUVEC cells, as primary normal cells, showed xanthanolides as the potent cytotoxic compounds at low doses compared to stigmasterol by inducing cells cycle arrest, apoptotic cell death and an inhibitory effect on protein kinase activity. Moreover, xanthinosin and xanthanol exhibited selective inhibitory effect on cancer cells with significantly less cytotoxicity against normal HUVEC cells [148]. The hydro-alcoholic extract of the fruit induced dose-dependent cell death against the HeLa cell line by altering the intracellular antioxidant levels via inhibition of the antioxidants glutathione, SOD, and catalase, with a subsequent increase in lipid peroxidation levels [149]. In another study, among three newly isolated ent-kauranoid glycosides from the fruits of *X. strumarium*, fructusnoid C showed anticancer activity in cancer cells with an IC_50_ in the range of 7.6 to >50 μM [150].

According to reports, *X. strumarium* has medium to strong allergenic effects and induces severe toxicity in mammals due to the presence of toxic phytochemical like sulphated glycoside and carboxyatractyloside [144,145,151]. Recent studies have also found that the extracts of *X. strumarium* have toxic effects. One study reported that the LD_50_ for *X. strumarium* fruit was 155.93, 317.80, and 167.6 g/kg body weight of mice administrated with processed water extract, processed ethanol extract, crude water extract, and crude ethanol extract, respectively, whereby water extract induced greater acute toxicity than ethanol extract. Mice given water extract intragastrically displayed toxic symptoms, such as cyanosis, intention tremor, respiratory inhibition, loss of righting reflex, convulsion, and repose. The intragastric administration of ethanol extract to mice resulted in the toxic symptoms of repose, abdominal respiration, intentional tremor, intermittent convulsions, and incontinence [152]. Administration of ethyl acetate, n-butanol, and water extract of fruits (0.06, 0.3, and 0.7 g/kg for 28 days) resulted into hepatotoxicity in rats evidenced from pathological alterations like enlarged hepatic cell space, karyolysis, and inflammatory cell infiltration [153]. Two kaurene glycosides (atractyloside and carbxyatractyloside) isolated from the fruits induced oxidative stress, which causes lipid peroxidation in the liver, causing severe hepatotoxicity in mice, which were administrated intraperitoneal with both compounds in the concentration range of 50 to 200 mg/kg body weight for 5 days [154]. The rats treated via intragastric administration of 30.0 g/kg with the water extract of fruit for 5 days induced hepatocyte injury by influencing the fatty acid metabolism. This was reflected in the increased plasma levels of low-density lipoprotein (LDL), β-HB, glutamate, choline, lactate, acetate, and acetone, while the valine and glucose levels were decreased indicating that the toxicological mechanism is linked to lipid and energy metabolism [155]. Apart from hepatoxicity, the extracts of this plant also induced neurotoxicity by significantly depressing the central nervous system. Administration of methanol extracts of the aerial parts of *X. strumarium* in doses of 100, 200, and 300 mg/kg resulted in altered general behavioral patterns, decreased spontaneous motility, prolonged pentobarbitone-induced sleep, suppressed exploratory behavioral patterns, and situational avoidance [156]. Additionally, hydroalcoholic extracts of the aerial parts of *X. strumarium* (25–100 g/mL) significantly reduced CHO cell viability and induced DNA damage at cytotoxic concentrations via sister chromatid exchanges and chromosome aberrations [157]. A case study further reported that intoxication occurred in a healthy young woman after consumption of a herbal preparation of *X. Strumarium*, which resulted in hepatic damage, symptomatic hypoglycemia, and seizures [158].

### 3.3. Withania somnifera *(L.) Dunal*

*W. somnifera* (poisonous gooseberry), belonging to the Solanaceae family, is one of the most popular and important medicinal plants in Ayurvedic medicine worldwide and is widely used as aphrodisiacs, diuretics, and for treating memory loss [159]. This species is diversely distributed throughout the world, including Africa. In Africa, this plant is indigenous in several countries, including Congo, Djibouti, Egypt, Lesotho, Liberia, Mali, Nigeria, South Africa, South Sudan, Sudan, Swaziland, and Tanzania [160]. The plant is a small shrub that grows up to 2 m tall and 1 m wide with the majority of the plant being covered in short, fine, branched, and silver–grey hairs. When ripe, the fruit is an orange–red to red, spherical, and hairless berry that is 5–8 mm across and enclosed by an enlarged calyx, with numerous kidney-shaped, 2.5 mm across, and very light brown seeds. Fruiting time is mostly from October to July [161]. Traditionally, different parts of this plant have been used for the treatment of eye infections, cuts, wounds, abscesses, inflammation, kidneys disorders, fever, skin infection, measles, gynecological problems, hemorrhoids, pulmonary troubles, asthma, rheumatism, cardiovascular disorders, Alzheimer’s disease, neurocognitive disorders, diabetes, arthritis, and malaria. This plant also has other properties like abortifacients, sedative, and aphrodisiac [160].

In one study, a phytochemical analysis of petroleum ether and acetone extracts of *W. somnifera* fruit showed the presence of coumarins (scopoletin and aesculetin), phytosterols (stigmasterol and sitosterol), steroidal lactones of the withanolides (5β,6α,14α,17β,20β-pentahy-droxy-1-oxo-20S,22R-witha-2,24-dienolide and 6α,7α-epoxy-5α,14α,17α,23β-tetrahydroxy-1-oxo-22R-witha-2,24-dienolide), and triterpene (β-amyrin) [162]. While in another study, the presence of withanolide alkaloids and unique serotonin conjugates of withanamides was reported [163]. Further, several different unsaturated fatty acids and withanolides were also isolated from *W. somnifera* seeds [163]. Although most of these extracted phytocompounds from fruits have been investigated for their biological activities, like antioxidant and antimicrobial properties, not much has been explored for their anticancer activity. A study conducted by Abutaha exhibited antineoplastic properties of a methanolic extract of the unripe fruit of *W. somnifera* against several human cancer cells by inducing apoptotic cell death. Further, the study also demonstrated anticancer properties of different solvent fractions isolated from *W. somnifera* crude extract of the fruit, showing an IC_50_ value of 95 µg/mL against HepG2 cells in a dichloromethane fraction [164]. As per certain reported studies with other aerial parts of *W. somnifera* rich in withanolides showing anticancer properties, these alkaloids are probably responsible for the anticancer properties of the fruit extract, as mentioned in the above study. For example, withanolide extracted from the leaves of *W. somnifera* have been reported to inhibit both inducible and constitutive NF-κB activation, thus being involved in blocking the NF-κB activation pathway and NF-κB-regulated gene products that control inflammation, tumor cell survival, invasion, osteoclastogenesis, and potentiating apoptotic cell death in cancerous cell lines [165]. In an interesting study conducted by Oza et al., for the first time, a plant-based L-asparaginase enzyme with antitumor activity was reported from *W. somnifera* extract. The purified enzyme was characterized as a homodimer with a molecular weight of 72 ± 0.5 kDa, which showed strong cytotoxic properties against leukemia cells and is considered a promising anticancer agent in the therapy of certain types of lymphoma and leukemia [159].

A toxicology study showed the administration of 2000 mg/kg body weight of the hydroalcoholic *W. somnifera* root extract for acute and subacute oral toxicities to be practically safe in Wistar rats [166,167]. Alcoholic root extracts from *W. somnifera* were tested for their acute (24 h) and subacute (30 day) toxicity in Wistar rats and Swiss albino mice. The calculated LD_50_ value was 1260 mg/kg body weight. Repeated administration of 100 mg/kg for 30 days, resulted in a significant reduction in the weight of the spleen, thymus, and adrenals in Wistar rats [168]. *W. somnifera* whole plant hydromethanolic extract given orally to Sprague–Dawley rats for 28 consecutive days at doses of 100, 300, and 1000 mg/kg/day was determined to be safe up to a dose of 1000 mg/kg/day [169]. Another study reported the evaluation of *W. somnifera* capsule’s (aqueous extract: 8:1) activity, safety, and dose-related tolerability in healthy individuals. The administration of capsules in daily dosages of 750 mg/day for 10 days, 1000 mg/day for the next 10 days, and 1250 mg/day for the last 10 days was well tolerated in volunteers. Hematological and biochemical tests on the health of the organs concluded that the formulation was safe. The formulation also revealed muscle strengthening, lipid lowering, and improved sleep quality [170]. A systematic review, including a total of 69 (39 preclinical and 30 clinical) studies, showed the root of *W. somnifera* as a promising, safe, and effective traditional medicine for treating schizophrenia, chronic stress, insomnia, anxiety, memory/cognitive enhancement, obsessive-compulsive disorder, rheumatoid arthritis, type-2 diabetes, and male infertility. It also has fertility promotion activity in females, adaptogenic, growth-promoter activity in children, and acts as an adjuvant for lowering fatigue and improving quality of life among cancer patients undergoing chemotherapy [171].

### 3.4. Xylopia aethiopica *(Dunal) A. Rich*

*X. aethiopica* (English African pepper) which grows as a tall, slim, and aromatic evergreen tree in lowland rainforests and moist fringe forests in the Savanna region of Africa belongs to the Annonaceae family. This plant is widely distributed in West, East, and Southern Africa, including Angola, Cameroon, Congo, Central African Republic, Ethiopia, Ghana, Kenya, Malawi, Mozambique, Nigeria, Senegal, Tanzania, Uganda, Zambia, and Zimbabwe. This tree that can reach heights of 15 to 30 m and has a narrow crown with many branches. The clear, straight stem, which can range in diameter from 25 to 70 cm, frequently has short buttresses or prop roots. It produces fragrant fruits in bouquets of 12–20 bacciferous-looking capsules arranged in capitula and consist of slender, slightly curved pods with approximately 15 carpels [172,173]. Because of its numerous therapeutic applications, this plant has been essential in traditional medicine to treat a variety of conditions, including boils, sores, wounds, and cuts, as well as cough, biliousness, bronchitis, rheumatism, dysentery, malaria, uterine fibroid, amenorrhea, and more [173,174]. There are numerous documented uses for the fruit of *X. aethiopica* in folkloric medicine in nations like Ghana and Nigeria, where it has traditionally been used to treat a variety of conditions, including amenorrhea, cough, diabetes, dysentery, female infertility, hemorrhoids, malaria, syphilis, uterine fibroid, and several other ailments [175,176].

As per the ethnobotanical survey, fruits and seeds of *X. aethiopica* have a long history of use in herbal concoctions as treatment options for prostate, breast, and cervical cancer in African countries like Nigeria, Ghana, and Gabon [177]. Bioassay-guided fractionation showed a rich source of isolated diterpenoids, alkaloids in the fruit extracts, and several different amides and lignanamides in the seed extracts of *X. aethiopica* [178]. The treatment of various cell lines with the methanol extract of *X. aethiopica* fruits led to a dose-dependent growth inhibition mediated by cell cycle arrest at the sub-G0/G1 and G2/M phases along with activation of mitochondrial apoptotic cell death due the upregulation of the p53 and p21 genes and an increase in the Bax/Bcl-2 ratio [179]. Similarly, ethanol extract *X. aethiopica* fruits induced significant cell toxicity in cancer cells mediated by caspase-dependent apoptosis by modulating mitochondrial bioenergetics [180]. In one report, ent-15-oxokaur-16-en-19-oic acid, isolated as an active anticancer phytochemical from *X. aethiopica* ethanol extract, showed promising cytotoxic activity against a panel of cancer cell lines by triggering DNA damage resulting from cell cycle arrest in the G1 phase followed by apoptotic cell death [181].

Kuete et al. reported the results of an investigation of the antiproliferative efficacy of methanol crude extract of the fruits of *X. aethiopica*, showing strong inhibitory effects against both drug-sensitive and -resistant cells at a lower dose of >10 mg/mL. As an anticancer agent, this extract further showed cytotoxicity against human umbilical vein endothelial cells (HUVECs) at a very high concentration of >80 mg/mL (EC_50_). As an interesting fact, this extract further showed antiangiogenic effects in a chicken chorioallantoic membrane assay model with an inhibition of vascularization by 47.53% at a concentration of 20 mg/mL [182]. The authors further extended the study to investigate the antiproliferative effect of this extract on several sensitive and drug-resistant leukemia, breast, and glioma cancer cell lines. The tested extract showed the highest IC_50_ dose of approximately 30 mg/mL, even with MDR cell lines, by inducing cancer cell growth inhibition mediated through cell cycle arrest between the G0/G1 and S phases, loss of MMP, and enhanced ROS production [183]. The low IC_50_ value of the tested extract against all cancer cells promoted the authors to isolate the bioactive phytochemicals. Therefore, in an extended study, the anticancer effects of several phytocompounds, such as 16α-hydroxy-ent-kauran-19-oic acid; 3,4′,5-trihydroxy-6″,6″-dimethylpyrano[2,3-γ]flavone; isotetrandrine; and trans-tiliroside, isolated from methanol crude extract of *X. aethiopica* fruits were studied against several drug-sensitive and multidrug-resistant (MDR) cell lines. Among all of the isolated compounds, 3,4′,5-trihydroxy-6″,6″-dimethylpyrano[2,3-g]flavone and isotetrandrine showed the most effective anticancer property, even for MDR cancer cells where the mode of action for the first active constituent was reported because of the induction of apoptosis mediated by MMP disruption, while the second compound induced apoptosis mediated by increased ROS generation [184]. This study showed the need for the investigation of several different plant-based phytochemicals for their potential as antiproliferative agents against multifactorial-resistant cancer types. Furthermore, in another study, the antiproliferative activity of *X. aethiopica* fruit essential oil was also reported against the Hep-2 cancer cell line [185]. A study conducted by Ribeiro et al. broadened the chemical profile of *X. aethiopica* fruit hydro ethanol extract with the identification of six cinnamoylquinic acid derivatives and twenty-four flavonoid glycosides determined using HPLC-DAD-ESI (Ion Trap)-MSn and UPLC-ESI-QTOF-MS2 analyses. The tested extracts induced cancer cell death by activating caspase-3 [186].

Although it is widely used in conventional medicine and a significant number of in vitro and animal studies have been conducted to support its therapeutic uses, little research has been performed to determine the safety or toxicity of *X. aethiopica*. Oral administration of *X. aethiopica* fruit ethanol extract showed an LD_50_ of 3464 mg/kg body weight in albino rats [187]. The bioactivity of the hydroalcoholic extract of *X. aethiopica* leaves on larvae was monitored using the brine shrimp lethality test in the concentration range of 0.049 mg/mL to 25 mg/mL with an IC_50_ of 0.64 mg/mL. An acute toxicity study of this extract upon oral administration in Wistar rats showed an LD_50_ > 5000 mg/kg body weight. Further, no adverse effects in rats were observed after subchronic treatment at 500 and 1000 mg/kg doses for 28 days [188]. An acute toxicity study with an aqueous fruit extract of *X. aethiopica* (Dunal) A. in albino rats showed an LD_50_ dose of 2154 mg/kg. Rats treated with 1000 and 1600 mg/kg of extract showed clinical signs of toxicity, such as loss of appetite, piloerection, stretching of the abdomen, one-sided movement, diarrhea, and lethargy. Administration of high doses of 2900 and 5000 mg/kg led to death of the rats. Histological examination of tissue samples from the liver, kidney, and spleen revealed vascular obstruction, glomerulus architecture that deteriorated along with renal tubule distortion, and severe hemosiderin in each of the aforementioned organs [189]. Hexane, methanol, and extract of *X. aethiopica* leaves induced kidney and liver damage in Wistar rats treated at 400 mg/kg body weight [190].

### 3.5. Abelmoschus esculentus *(L.) Moench*

*A. esculentus* (okra), which belongs to the Malvaceae family, is an annual shrub that is commonly cultivated throughout the tropics and warmer temperate regions of the world and is a very popular vegetable in subtropical Asia and Africa [191]. The plant is a stout, erect, and annual herb that grows up to 4 m tall. The okra fruit or pod is a six-chambered, fibrous-textured, and greenish capsule with a length of 10 to 30 cm and a diameter of 1 to 4 cm. It is slightly curved and tapers to a blunt point [192].

Okra has been used in folkloric medicine in many parts of East Africa to treat kidney stones, diabetes, jaundice, gastritis, gastric ulcers, and other infections or ailments. Phytochemical analysis has shown that okra is a rich source of several potent bioactive components, such as catechin; epigallocatechin; isoquercitrin; rhamnogalacturonan; quercetin-3-O-sophoroside; quercetin-3-O-[glucosyl(1->6) glucoside]-7-O-rhamnoside; (3β,21β)-19,21-epoxylup-20(29)-en-3-yl acetate; (3β)-9,18-dihydroxyolean-12-en-3-yl acetate; polyphenolic groups of compounds like flavonoids; hydroxycinnamic derivatives; 5,7,3′,4′-tetrahydroxy flavonol-3-O-[β-d-rhamnopyranosyl-(1→2)]-β-d-glucopyranoside’ rutin; procyanidins B1 and B2; 5,7,3′,4′-tetrahydroxy-4″-O-methylflavonol-3-O-β-d-glucopyranoside; 5,7,3′,4′-tetrahydroxy flavonol-3-O-[β-d-glucopyranosyl-(1→6)]-β-d-glucopyranoside14; and oligomeric proanthocyanidins [193,194,195]. Most of these bioactive compounds have been shown to induce potent anticancer effects in several studies [24,193,196].

Taiwo et al., for the first time, showed the presence of oleanene glucoside as oleanene skeleton triterpenoid and two quercetin glucosides as isoquercitrin and quercetin diglucoside isolated from the methanol extract of okra fruit. As an interesting finding, only oleanene glucoside showed mild toxicity towards cancer cells, followed by isoquercitrin which induced cytotoxicity only at a 100 μg/mL concentration, while quercetin diglucoside was completely nontoxic. However, when these compounds were tested for their ability to protect against ROS induction, isoquercitrin and quercetin diglucoside showed the most potent antioxidant property with IC_50_ values of 2.0 ± 0.8 μg/mL and 1.5 ± 0.4 μg/mL, respectively, with no protective effect induced by oleanene glucoside [194]. In another study, the anticancer property of extracts of okra pods was reported due to the presence of phenolic and terpenoid compounds, as well as certain volatile compounds like β-caryophyllene, limonene, and eugenol [197]. Another study showed flavonoid compounds including isoquercitrin present in the okra seed extract, which had significant cytotoxic effects against cancer cells when delivered in its native form, as well as in the form of polymeric micelles. The plausible mode of action was suggested to be via the inhibition of VEGF production leading to migration impairment, inducing apoptotic cell death [198]. Additionally, *Abelmoschus*-derived hyperin (quercetin-3-O-β-d-galactoside), an important flavonol glycoside phytocompound, was shown to induce strong anticancer potential against gastric cancer cells at a concentration of 50 and 100 µg/mL by targeting the Wnt/β-catenin signaling pathway which, in turn, inhibited cancer cell proliferation, migration, and invasion in both an in vitro and in vivo xenograft tumor mice model [199].

Other than the abovementioned phytochemicals with cytotoxic potential, a lectin isolated from okra seed meal, *A. esculentus* lectin (AEL), present as a 10.29 kDa monomer or as a 20.58 kDa dimer protein, also demonstrated selective cytotoxic effects against MCF-7 cells compared to normal skin fibroblast cells. AEL induced cell death via an apoptotic pathway, which was evident from the increased expression of pro-apoptotic caspase-3, caspase-9, and p21 genes, as well as enhanced Bax/Bcl-2 ratio [200]. Another study also revealed the antiproliferative and antimigratory effectiveness of AEL against neural cancer. The results showed the ability of AEL to induce G0/G1 phase cell cycle arrest and apoptosis by modulating caspase 3 and 7 expression, with a plausible association with enhanced intracellular ROS generation. As another interesting finding, this study further showed AEL-mediated downregulation of the circadian *clock* and *Bmal*1 gene expressions, which promote cancer cell growth and proliferation, therefore playing a significant role in glioma development [201]. Furthermore, cerium oxide (CeO2) nanoparticles synthesized from the fruits of *A. esculentus* induced significant cytotoxicity against HeLa cancer cells in a dose-dependent manner with an IC_50_ value of 85.74 µg/mL [202].

Toxicological studies with distilled water, hot water, and hot buffer (HB) of okra pod extract showed an LD_50_ of >5000 mg/kg body weight in the oral acute toxicity study. No toxicity was observed in a subchronic study with 1000 mg/kg extract ingested by mice for 28 days, which indicates the safety of okra extract for short-term administration [203]. Another study showed a value of LD_50_ > 2000 mg/kg as a safe dose in an acute oral toxicity study, whereby mice were treated with an aqueous and methanolic extract of *A. esculentus* seeds [204]. An acute oral toxicity assessment of *A. esculentus* peel and seed extracts in Wistar rats at 2 g/kg showed no signs of toxicity [205]. The few clinical studies of okra and its components on their antidiabetic and lipid-lowering effects did not report any negative side effects associated with the consumption of okra preparations [206,207,208,209]. Unfortunately, even widely used as a dietary fruit, clinical studies on the efficacy and induced toxicity concerning okra are inadequate.

### 3.6. Carissa macrocarpa *(Eckl.) A.DC*.

*C. macrocarpa* (Natal plum) from the Apocynaceae family, which is native to Central and Southern Africa, grows as coastal bush widespread along the east coast of South Africa, from Humansdorp, KwaZulu-Natal to Kosi Bay and extending to Mozambique [210]. This plant is a fast-growing shrub that usually grows along the coast. It typically grows as a dense, prickly shrub, but it can also develop into a small tree up to 4 m tall. The plant yields large, oval, and fleshy red ripe edible fruits, approximately 15 mm long, enclosing a firm reddish pulp in which numerous small seeds are embedded [113,210].

In many parts of Southern Africa, the fruits of this plant have traditionally been used to treat certain symptoms in human immunodeficiency virus (HIV)- and hepatitis-affected patients [118,119]. Phytocompounds, like β-amyrin, methyl oleanolate, and 3β-hydroxyolean-11-en-28,13β-olide, have been isolated from dichloromethane extract and oleanolic acid from the methanol extract; amongst them oleanolic acid and β-amyrin have been suggested to possess anticancer activities [118]. Souilem et al. evaluated the phytochemical profile of hydroethanolic extract of *C. macrocarpa* fruits with high contents of sugars and polyunsaturated fatty acids, five organic acids (oxalic, quinic, shikimic, ascorbic, and citric acid) and two tocopherol isoforms. This extract demonstrated a significant inhibitory effect against cancer cells with a GI_50_ in the range of 52.1 to >109 μg/mL, without toxicity for nontumor cells [119]. An acute oral toxicity study conducted on Wistar rats orally administered with *C. macrocarpa* MeOH fraction of the leaves showed no toxicity, mortalities, or any physical or behavioral abnormalities at the highest dose of 500 mg/kg body weight [211].

### 3.7. Carpobrotus edulis *(L.) L. Bolus*

*C. edulis* (sour fig) is a robust, evergreen, succulent, drought-resistant, and trailing perennial halophyte plant that belongs to the Aizoaceae family, which is a native to the Cape Coast region of South Africa but have become one of the most important invasive species in Europe, Australia, and the United States [212]. The plant is a sturdy, flat-growing, and trailing perennial with roots that form dense mats at nodes and horizontal stems that curve upwards at the growing point. The plant yields edible fruits that measure 35 mm in diameter and are indehiscent and shaped like spinning tops with the seeds enmeshed in the mucilage. When ripe, the fruit turns yellow and becomes fragrant. With time, the fruit’s exterior wall turns leathery, wrinkly, and yellowish [81].

Different parts of this succulent have been used in traditional medicine across South Africa to treat a wide range of ailments, including microbial infections, tuberculosis, respiratory infections, skin problems, sore throat and mouth infection, HIV/AIDS, hypertension, and diabetes mellitus. [212,213]. The phytochemical analysis of different extracts of the peel and flesh of fruits of *C. edulis* showed the highest amounts of phenolic acids, flavonoids, and coumarins in peel extracts. Further, the HPLC-ESI-MS/MS analysis revealed the presence of specific phytocompounds in a particular fruit part, for example, coumaric acid and uvaol in the peel and vanillin and kaempferol-O-(rhamnosyl)hexosylhexoside in the flesh, and the presence of azelaic acid and emodin was also reported for the first time in *C*. *edulis* fruits [212]. Many of these phytochemicals hold potential as an effective anticancer agent; unfortunately, no studies have been reported that evaluated the same but with fruits of *C. edulis*. Castañeda-Loaiza et al. demonstrated that the fruit extracts have insignificant to low cytotoxicity on the human keratinocyte cell line HaCat due to the antioxidant properties and tyrosinase inhibition activity, indicating its potential exploitation as a safer cosmetic ingredient [212].

In one study, the brine shrimp lethality test revealed that a leaves extract of *C. edulis* was relatively less toxic, with death rates of 47.43% and 48.06% for 24 h and 48 h, respectively [214]. This finding was further supported by another study that found that aqueous and methanolic extracts of *C. edulis* at a 2 mg/mL concentration were safe or nontoxic using a brine shrimp lethality bioassay [215]. A study conducted with Sprague–Dawley rats showed that aqueous extracts of the leaves of *C. edulis* are potentially safe with an LD_50_ dose of >2000 mg/kg. Further, in an subacute toxicity study, the rats’ body weight, feed and water intake, histology, or the tested biochemical parameters did not show any negative effects upon oral administration of 1000 mg/kg extract for 28 days [216].

### 3.8. Syzygium cumini *(L.) Skeels*

*S. cumini* (java plum) is a large evergreen tropical tree belonging to the Myrtaceae family and is mainly native to the Indian subcontinent; however, today this tree is cultivated in many parts of Africa, including Eastern Africa, South Africa, and Madagascar. *S. cumini* is a large, densely foliated evergreen tree 5–10 m tall and has thick, woody scale-exfoliating, and greyish–brown bark. The fruits are sweetly flavored, dark-purple or nearly black berries that are oblong and frequently 1.5 to 3.5 cm long. They are luscious, fleshy, and edible, with one large seed inside [217].

Because of the presence of bioactive compounds in various plant parts, *S. cumini* is known to have a wide range of medicinal properties. The fruits are used to treat pharyngitis and splenic diseases, while the bark is used as an astringent, an anthelmintic, and a carminative. The leaves are used to treat dermopathies, gastropathies, constipation, leucorrhea, and diabetes. Additionally, seeds are employed in the treatment of diabetes and as astringents and diuretics [217,218]. In Indian traditional medicine, the use of *S. cumini* seeds have shown great potential in the treatment of diabetes, scurvy, hypertension, and diarrhea. The pulp part of this fruit has been reported to be rich in anthocyanin content, whereas its seed contains high amounts of ellagitannins and ellagic acid, which is responsible for its health benefits [39,219]. The presence of such potent anticancer bioactive phytochemicals also showed the potential of *S. cumini* fruits as an antineoplastic and chemopreventive agent [220]. In one study, hydrolyzed pulp and seed extracts of java plum showed strong antiproliferative activity against cancer cells compared to unhydrolyzed extracts, which was attributed to the high content of anthocyanidins, polyphenolics, ellagitannins, ellagic acid flavonoids, and terpenes [219]. Further, a standardized *S. cumini* fruit pulp extract that reported high contents of five anthocyanidins, delphinidin, cyanidin, petunidin, peonidin, and malvidin, in the form of diglucosides exhibited concentration- and time-dependent antiproliferative and pro-apoptotic effects against estrogen-dependent/aromatase-positive and estrogen-independent triple-negative breast cancer cells. Importantly, the effects were selective and more pronounced in estrogen-dependent/aromatase-positive cells, with a negligible effect on normal nontumorigenic breast cell lines [221]. The crude and methanolic extracts of java plum fruit skin along with the outermost layer demonstrated antiproliferative and apoptotic effects against HPV-18-positive HeLa and HPV-16-positive SiHa cervical cancer cell lines [222].

Interestingly, the anticancer efficacy of *S. cumini* seed hydroalcoholic extract was investigated against chemical carcinogen-induced skin carcinogenesis in Swiss albino mice tumor models. The study showed that daily oral administration of extract at a dose of 125 mg/kg body weight resulted in a significant reduction in the number of skin papillomas along with a greater decrease in tumor burden, tumor yield, and tumor incidence (75%). The effect was suggested due to the strong antioxidant properties of anthocyanins, flavonoids, gallic acids, quercetin, tannins, etc., present in the extract. This demonstrated the utility of the extract both as a cancer chemopreventive and as a treatment agent [223]. Similarly, the extract also showed antitumor and antioxidative efficacy against an in vivo gastric carcinogenesis model, with a significant reduction in tumor incidence, tumor burden, and cumulative number of gastric cancer papillomas, whereby the Swiss albino mice were orally administrated an extract dose of 25 mg/kg of body weight per day. The efficacy was due to their potential to reduce the lipid peroxidation level along with significant enhancement of the antioxidant protein GSH and catalase activities [224].

According to several studies, orally administered *S. cumini* does not cause acute or chronic toxicity [218,225]. An acute toxicity study with ethanolic extract of *S. cumini* seeds indicated an LD_50_ of >2000 mg/kg in Albino mice, while a subacute toxicity study at a given dose of 1000 mg/kg, once daily for 4 weeks, indicated no significant change in the general physiological and hematological parameters of liver and renal functioning [226]. The LD_50_ dose for a methanolic extract of seeds was found to be 5000 mg/kg bodyweight in albino rats [227]. As per a study conducted by Silva et al., the oral administration of a hydroalcoholic extract of *S. cumini* leaves at doses up to 2 and 6 g/kg to rats and mice, respectively, did not have an immediate or long-lasting toxic effect up to 14 days, while intraperitoneal administration of the extract killed 70% of the mice at a dose of 0.5 g/kg and 67% of the rats at a dose of 2 g/kg [228]. Additionally, the fact that the LD_50_ value of the methanolic extract of the fruit was above 3000 mg/kg indicates that traditional fruit is practically safe or nontoxic, because no behavioral changes and no mortality were noted during acute (14 day) and subacute (28 day) toxicity studies in rats [229]. Moreover, clinical studies on the antihyperglycernic effect of *S. cumini* also supported their safe use in humans [218,230,231,232,233].

### 3.9. Kigelia Africana *(Lam.) Benth*.

*K. africana* (sausage tree), which belongs to the Bignoniaceae family, is a tropical African medicinal plant that is widely cultivated in Central, Southern, and Western Africa. The plant is a medium to large semi-deciduous tree with a short, squat trunk that has light brown, occasionally flaky bark and supports a dense, rounded to spreading crown of leathery, slightly glossy, and deciduous foliage that is 18 m high and 20 m wide. Its fruits are long and woody, resembling sausages, and they hang from the tree on long stalks that resemble cords. The enormous size of the fruit can reach 1 m × 18 cm and weigh up to 12 kg, and fruiting season is from December to June. When ripe, the fruits are brownish-grey in color and contain a tough, seed-filled pulp that is inedible [234,235].

This tree has been reported to be used as traditional folk medicine in the treatment of a variety of skin conditions, impetigo, syphilis, leprosy, skin cancer, dysentery, malaria, diabetes, pneumonia, worm infestations, convulsions, venereal diseases, toothaches, or as a snakebite antidote [236]. Phytochemical investigation of this fruit’s extracts have shown the presence of iridoid glycosides, derivatives of furanone, phenylpropanoid and eucommiols, naphthoquinones, flavonoids, terpenes, terpenoids, coumarins, and phenylethanoglycosides [235,236,237,238]. In several African countries, the fruit extracts of *K. africana* have been used traditionally as herbal remedies for the treatment of cancer-related infections, melanoma, skin neoplasms, and endometrial cancer [235,239].

Detailed studies show that several phytocompounds, like norviburtinal, demethylkigelin, kigelin, furonaphthoquinone, 2-(1-hydroxyethyl)-naphtho[2,3-b]furan-4,9-dione, and ferulic acid, present in the fruit extract fractions are some of the major cytotoxic phytocompounds against cancer cells [240,241]. Even the oil obtained from *K. africana* seeds have shown to inhibit cancer cell growth [242]. In vivo studies further demonstrated the effectiveness of the fruit extract on forestomach tumorigenesis mice model, whereby 67% and 76% inhibitions were reported in tumor incidence and burden on oral administration of the extract at a dose of 2 mg/day [243]. In another study, *K. africana* fruit extract was shown to be an effective anticancer agent in reversing the leukemic effects in a rat tumor model by alleviating the anemia indices and reducing the leukocytosis compared to untreated leukemic animals. Here, the animals were administered with 100 mg/mL of extract mixture containing 0.5 mL stem–bark, 0.5 mL fruit, and 0.2 mL leaf extract [244].

An in vitro study with water and/or dichloromethane:methanol extract of leaves was shown to inhibit cell growth in human kidney epithelial cell line by 22% and 16%, respectively [245]. The ethanolic root extract of *K. africana* toxicity revealed a lethality-testing effect on brine shrimp larvae in a rose dose-dependent manner with LC_50_ and LC_90_ values of 100 and 350 μg/mL, respectively [246]. Only a few toxicity studies on various *K. africana* extracts have been reported, and the majority of these studies focused on acute toxicity [235]. Oral administration of hydromethanolic root extracts of *K. africana* indicated an LD_50_ dose of 5000 mg/kg body weight in albino rats, while a 3000 mg/kg dose of the extract was toxic at subchronic administration for 28 days, as indicated by liver and kidneys lesions [247]. Another study on the oral administration of ethanolic bark extract showed an LD_50_ of 955 mg/kg body weight [246]. The study of the acute oral toxicity of the leaves’ methanolic extract showed no behavioral changes, toxic reaction, or mortality in mice up to 2500 mg/kg bodyweight, while a dose of 5000 mg/kg resulted in death and the development of toxic symptoms [248]. Oral acute toxicity study with methanolic stem–bark showed no toxicity at lower doses (10 mg/kg, 100 mg/kg, and 1000 mg/kg) while higher doses (1600 mg/kg, 2900 mg/kg, and 5000 mg/kg) resulted in the treatment animals’ deaths. The calculated LD_50_ was then determined to be 1264.9 mg/kg body weight [249]. The LD_50_ of the intraperitoneally administered aqueous leaf extract to mice was calculated to be 785.6524 mg/kg [250]. Another study found that the intraperitoneal administration of stem–bark methanol extract had a somewhat higher LD_50_ of 3000 mg/kg in mice [251].

### 3.10. Annona muricata *L*.

*A. muricata* (soursop), belonging to the Annonaceae family, is native to the warmest tropical areas in South and North America but now widely grows throughout tropical and subtropical parts of the world, including the rainforests of Africa. The tree grows to a height of 5–8 m and a diameter of 15–83 cm, with a large, glossy, and dark-green leafy canopy, and it typically produces flowers and fruit throughout the year. Its green fruits are 15–20 cm in diameter, edible, and heart shaped with 55–170 fresh black seeds that turn light brown when dried. The flesh is white and creamy and has a distinctive flavor and aroma [252].

Several parts of this plant have been used as traditional medicine for the treatment of several ailments, including cystitis, diabetes, headaches, skin diseases, vaginal infections colds, flu, asthma, malaria, rheumatism, kidney disorders, insomnia, and many more [252]. Several bioactive constituents are present in the whole fruit, pericarp, and seeds of *A. muricata*, including annonaceous acetogenins (metabolites and products of the polyketide pathway), alkaloids, flavonoids, and sterols. These bioactive compounds are suggested to be responsible for their anticancer properties [253]. The crude ethyl acetate extract of *A. muricata* pericarp showed significant cytotoxicity against the cell line U-937 [254]. C-35 annonaceous acetogenins (muricins M and N) and C-37 annonaceous acetogenins isolated from bioassay-guided fractionation of this fruit exhibited cancer cell growth inhibition with the following potency: muricin N > muricin M > muricenin [255]. In another study, the authors investigated the anticancer potential of monotetrahydrofuran annonaceous acetogenins, (muricin H, muricin I, and cis-annomontacin), acetogenins, annonacin, annonacinone, annomontacin, murisolin, and xylomaticin isolated from the *A. muricata* seeds’ extracts, whereby monotetrahydrofuran annonaceous acetogenins showed potency [256]. A study conducted by Dai et al. demonstrated the selective inhibition of the growth of EGFR-overexpressing human breast cancer cells upon treatment with aqueous acetone fruit extract of *A. muricata*, while other breast cancer cells were less sensitive to the treatment, and no significant cytotoxicity was observed on nontumorigenic human breast epithelial cells. The treatment resulted in the downregulation of EGFR gene expression, G0/G1 cell cycle arrest, and induction of apoptotic cell death. The study further showed the effectiveness of the dietary treatment of the xenograft mouse model with a 200 mg/kg diet for 5 weeks, resulting in tumor growth inhibition by negatively regulating the EGFR/ERK signaling pathway [257].

The current information on the plant’s safety aspects shows the toxicology effect of the plant due to the presence of neurotoxic annaceous acetogenins and benzyltetrahydro-isoquinoline alkaloids [252,258]. The oral acute toxicity study showed an LD_50_ dose of 1.67 g/kg with an ethanol extract from *A. muricata* leaves in Wistar rats [259]. The aqueous extract of *A. muricata* leaves indicated to be safe with an LD_50_ dose of <5 g/kg body weight in Swiss albino mice; however, the extract at 1000 mg and above may likely cause kidney damage when administered for 14 days [260]. One study reported that among the other plant parts, the seeds, fruit pulp, and pericarp of *A. muricata* were the most toxic when extracted in methanol indicated by an LD_50_ dose of 0.85 g/kg, 1.09 g/kg, and 1.58 g/kg, respectively [261]. In Swiss albino mice, a single dose of 250, 500, or 1000 mg/kg of aqueous extract of leaves did not show any behavioral changes or death in experimental animals [262].

### 3.11. Persea americana *Mill*.

*P. americana* (avocado), a member of the flowering plant family Lauraceae, is native to Mexico and Central America. The plant was introduced in South Africa by settlers and colonies and, presently, they are cultivated in the humid, subtropical regions of the country, in the provinces of Limpopo and Mpumalanga. The avocado tree is a multistemmed tropical evergreen that typically reaches a mature height of 30 to 60 feet, and the tree canopy ranges from low, dense, and symmetrical to upright and asymmetrical. The fruit is a berry with a single large seed and a yellow-colored, buttery pulp surrounding it that can weigh up to 2.3 kg and range in shape from spherical to pyriform. The thickness and texture of the skin varies. Depending on the variety, the fruit can be green, black, purple, or reddish when it has reaches maturity [263,264].

**Figure 1:**
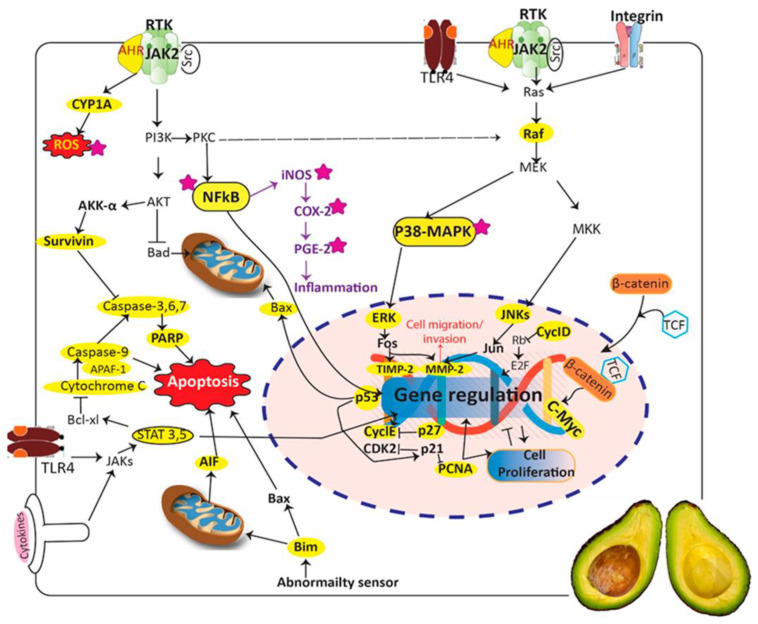
Anticancer molecular pathways targeted by *P. americana* extracts and isolated phytochemicals. Adapted from [267].

The plant parts are used in traditional medicine to treat a number of conditions, including skin rashes, diabetes, bronchitis, diarrhea, hypertension, stomach aches, menorrhagia, coughs, liver obstructions, menstrual problems, rheumatism, and high uric acid [263,265,266]. The fruits are rich in several different phytochemicals and metabolites, including fatty alcohols, furan derivatives, carotenoids, phenolics, diterpenoids, lignan derivatives, and essential oils. Several preclinical studies demonstrating the cytotoxic properties of avocado fruits against different types of cancer have been extensively reported and reviewed. Several bioactive phytocompounds, such as proanthocyanidins, aliphatic acetogenins, lutein, zeaxanthin, β-cryptoxanthin, α-carotene, β-carotene, α-tocopherol, γ-tocopherol, 1,2,4-trihydroxynonadecane, 1,2,4-trihydroxyheptadec-16-ene, 1,2,4-trihydroxyheptadec-16-yne, triterpenoid, 4-hydroxy-5-methylene-3-undecyclidenedihydrofuran-2 (3H)-one, (2R)-(12Z,15Z)-2-hydroxy-4-oxoheneicosa-12,15-dien-1-yl acetate, and persenone a and b, isolated from the whole fruit, pulp, and seeds of avocado have shown significant cancer cell inhibition in vitro and in vivo. Detailed studies have shown that these bioactive phytochemicals target several molecular targets involved in cancer cell proliferation, survival, migration/invasion, and apoptosis, as well as inflammatory responses (Figure 1) [267].

A case-control study suggested that higher intake of avocado in men is associated with reduced risk of prostate cancer. This fruit is a major source of dietary monounsaturated fatty acid (MUFA), where oleic acid itself accounts for ~50% of the MUFA content, which resulted in a high whole blood oleic acid proportion in the study population. However, the relationship between MUFA content in avocado and prostate cancer may be more complex and needs further investigation [268].

The ethanol extract of avocado flesh is regarded to be safe, as indicated by no toxic symptoms or lethality in Wistar rats, showing the maximal tolerable dose (MTD) to be ≥10 g/kg and an L_50_ of >5 g/kg body weight [269]. Similar results were also shown in another study, whereby the acute treatment of rats with an aqueous extract of seeds in doses above 10 g/kg did not result in death or any other toxicological symptoms [270]. An LD_50_ > 5000 mg/kg was reported in acute studies in Wistar rats treated with an ethanolic extract of seeds. However, subacute oral administration of extract at 400 and 800 mg/kg for 28 days significantly led to liver and kidney damage [271]. Another study determined the effect of avocado seed flour, whereby the oral LD_50_ was 1767 mg/kg body weight of mice [272]. Also an LD_50_ > 4000 mg/kg was tested in Sprague–Dawley rats treated with a methanolic extract of seeds with no reports of mortality or overt signs of toxicity or distress [273]. While a very high dose of 15,000 mg/kg body weight was indicated as the oral LD_50_ for an ethanol extract of avocado fruits [274].

The fruit pulp oil of *P. americana* showed no genotoxic effects in vitro both in V79 cells (highest concentration of 800 μg/mL) and in Swiss mice (highest concentration of 2000 mg/kg) using micronucleus assays. Additionally, both in vitro and in vivo, pulp oil indicated protection against other mutagen-induced genotoxicity. However, significant cytotoxicity was reported in V79 cells at concentrations of 1250, 2500, and 5000 μg/mL. Also, the long-term administration of a 1000 mg/kg dose indicated hepatic tissue damage, suggested due to the presence of high concentrations of palmitic acid [275]. Similar results were reported in another study, where chromosomal aberrations induced by cyclophosphamide in lymphocyte cell proliferation were reduced by methanolic extract of avocado fruits at a concentration of 200 mg/kg body weight of animal [276]. On the contrary, another study showed chromosomal aberrations in cultured human peripheral lymphocytes exposed to methanolic extracts of fruit and leaves, where the concentration ranged from 100 mg/kg, 200 mg/kg, and 300 mg/kg [277].

In one study, the administration of avocado leaves and isolated persin at a dose rate of 60–100 mg/kg demonstrated the development of noninfectious lesions in lactating mice’s mammary glands [278]. Several animal species, including sheep, goats, dogs, rabbits, ostriches, and horses, have been reported to experience avocado poisoning. For animals that are vulnerable, the leaves, fruits, and seeds of avocado trees are suggested to be toxic at high doses. Persin, an acetogenin derived from the biosynthesis of long-chain fatty acids, which is present in the leaves, fruits, and seeds of *P. americana*, is thought to be responsible for the toxic effects of avocado [279,280,281,282,283,284]. All of these studies suggest that the administration of P. *americana* supports its use as an ethnomedicine, while caution should be taken regarding the dose and long-term usage, as prolonged use may result in irreparable kidney and hepatic damage.

### 3.12. Punica granatum *L*.

*P. granatum* (pomegranate), belonging to the Punicaceae family, is a native shrub of the Mediterranean region. However, because of their adaptive behavior to a wide range of climates, from subtropical to Mediterranean, with cool winters and hot summers, they are grown worldwide. Being an exotic plant in South Africa, it is grown and cultivated throughout the country. Punica granatum is a spiny, deciduous shrub or small tree reaching heights of up to 4–5 m, with deep roots but slow growth, an open canopy, and a crown that branches from low down. The plant has flaky bark with thorny branches that wrap around one another. The edible fruit is a berry that is 5 to 12 cm in diameter, has a rounded hexagonal shape, thick reddish skin, and 600 or more seeds encased in a white, astringent, and spongy pulp. The edible aril is the fruit’s water-filled pulp that ranges in color from white to deep red or purple [285,286].

According to ancient Ayurvedic medicine and Chinese folk medicine, extracts and formulations made from the fruit, bark, juice, root, and dried peels have extensively been used over generations for treating ulcers, diarrhea, male infertility, diabetes, dysentery, intestinal parasites, malaria, bacterial and fungal infections, sore throats, coughs, urinary infections, digestive disorders, skin disorders, arthritis, and many more [287,288]. Among all of the reported fruits in this review, *P. granatum* is one of the most extensively researched fruits for its anticancer activities. Because of the richness of phytochemicals, like anthocyanins, tannins, flavonoids, and alkaloids bioactive compounds, the whole fruit, including peels, pericarp, seeds, and juice, have been studied, reported, and reviewed for their anticancer efficacy against several cancer types [285,288,289,290].

Interestingly, because of the promising efficacy of *P. granatum* fruits in targeting different cancer molecular pathways, several clinical studies have been initiated. Oral administration of the fruit extract (POMx) in prostate cancer patients for four weeks showed a significant accumulation of urolithin A, a *P. granatum* metabolite in the prostate. This was correlated with a significant decrease in an oxidative stress biomarker (8-hydroxy2′-deoxyguanosine) in prostate tissue [291]. In a phase II clinical study, patients with recurrent prostate cancer following primary therapy (surgery or radiotherapy) were treated with 8 ounces of pomegranate juice daily until disease progression. The results showed significant prolongation of prostate-specific antigen doubling time (PSADT) from an average of 15 months to 54 months following 33 months of therapy, along with a 23% increase in serum nitric oxide and significant reductions in oxidative state and sensitivity to oxidation of serum lipids post pomegranate juice consumption [292]. Further, a randomized, double-blind phase II clinical study showed a lengthening of PSADT by more than 6 months in prostate cancer patients following local therapy [293]. In another clinical study, patients with prostate cancer when administrated with 200 mL of pomegranate juice for 3 days showed the accumulation of ellagitannin metabolites urolithin A glucuronide, urolithin B glucuronide, and dimethyl ellagic acid in the prostate gland, responsible for its beneficial effects against prostate cancer [294].

The ethanolic extract of pomegranate seeds was considered to be safe in Swiss Webster mice at doses > 160 mg/kg body weight when administered as an oral topical solution without any induced acute systemic toxicity [295]. The intraperitoneal administration of a hydroalcoholic extract of fruits to mice indicated an LD_50_ dose of 731 mg/kg [296]. The use of methanolic extract of fruit peel showed no toxicity when administered directly via the oral cavity under 7.5 mg/kg, according to a 22-day gavage study with repeated doses in BALB/c mice [297]. Another study demonstrated an oral LD_50_ > 5 g/kg body weight and an intraperitoneal injection LD_50_ of 217 mg/kg body weight for rats and 187 mg/kg body weight for mice, respectively. No treatment-related toxicological changes were observed upon the administration of pomegranate extract [298].

A study conducted to assess the in vivo antigenotoxic and toxicological effects of fruit peel hydroethanolic extract exhibited a potent antigenotoxic effect in Swiss mice with significant reduction rates in doxorubicin-induced micronucleus frequency with 700 mg/kg treatment dose. Furthermore, no genotoxic or cytotoxic effects were detected in the mice [299]. Similar results were shown whereby leaf and fruit extracts (12.5, 25, 50, and 75 mg/kg) did not exhibit any cytotoxic effects on bone marrow cells in mice administered oral treatments for 10 days [300]. Additionally, mice given oral pomegranate aqueous extracts (1000 and 2000 mg/kg b.w.) had no genotoxic effect as measured using a micronucleus test [301]. The metabolites gallic acid (IC_50_ 41.2 μg/mL), ellagic acid, and punicalagin (IC_50_ 314.1 μg/mL) were found to be the most cytotoxic to bovine kidney epithelial cells (MDBKs) and were suggested to be the most toxic metabolites from a crude pomegranate fruit extract resulting in toxicity in farm animals [302]. Several clinical studies have looked into the effects of pomegranates on a wide range of medical disorders [303]. To fully establish the clinical applications and therapeutic role of pomegranate as an anticancer agent, additional carefully planned clinical trials are required.

Other than these reported fruits, as per the anticancer screening program report, other fruit extracts from Southern African plants like *Rhus lancea* E. Mey. ex Harv. & Sond. and *Gomphocarpus fruticosus* (L.) Aiton f. have shown moderate anticancer activity with total growth inhibition (TGI) at a concentration range of 13.23–16.66 mg/mL and 6.78–30.20 mg/mL, respectively [35]. Phytocompounds like 21-β-acetoxymelianone, 3-α-tigloylmelianol, and melianone isolated from the fruits of *Melia azedarach* have also been shown to induce moderate to strong cytotoxicity against cancer cells [304]. Aqueous extract of the fruit pulp and fractions of *Adansonia digitata* showed mild cytotoxic potential against human oral cancer cell lines [305]. In addition to these, there are several other native and non-native African plants, like *Rhus vulgaris* Meikle, *Carica papaya* L., *Solanum aculeastrum* Dunal subsp. *aculeastrum,* and *Capsicum frutescens* L., whose fruits and seeds have traditionally been used to treat or manage several cancer types [306].

**Table 2:** Summary of effects of fruit extracts or isolated phytochemicals against fruits and their phytochemical profiles.

Human Cancer type	Cell Lines and In Vivo Models	Phytochemicals/Fruit or Seeds Extracts	Effective Concentration	Reference
Colorectal cancer	HCT116, DLD-1	Extracts from *X. strumarium* and *T. terrestris* fruits	100 mg/mL	[129]
Oral cancer	SAS, TW2.6	Extracts from *X. strumarium* and *T. terrestris* fruits	50 and 100 mg/mL	[130]
Liver cancer	HepG2	Aqueous extract of *T. terrestris* fruits	200 and 500 mg/L	[131]
Colorectal, breast, liver cancer, and T-cell lymphoma	Colo20, HCT116, DLD, MCF-7, Jurkat, HepG2	*Withania somnifera* fruit extract	LC_50_ values of 287.8 (Colo20), 410.2 (HCT116), 226.3 (MCF7), 356.4 (Jurkat), and 164.7 (HepG2) µg/mL	[164]
Leukemia	Primary leukemia cells from patients	*W. somnifera* fruit extract	LD_50_ 1.45 ± 0.05 IU	[159]
Liver, lung cancer, fibrosarcoma, and T-cell lymphoma	HepG2, A549, Jurkat, HepG2	*W. somnifera* seed extract	Methanol extract LC_50_ values of 50.18 μg/mL (Jurkat), 112.9 μg/mL (HepG2), 163 μg/mL (L929) and ethyl acetate fraction LC_50_ 48.73 μg/mL (Jurkat), 68.739 μg/mL (HepG2), and 111 μg/mL (L929) Both extracts were less effective against A549	[146]
Liver, lung, and colon	HepG2, A549, HCT116, SW480	Isolated pentacyclic triterpenoids from the fruits of *Xanthium strumarium*	IC_50_ range between 4.27 mM and >100 mM	[147]
Cervical cancer	HeLa	Hydro-alcoholic extract of *X. strumarium* fruits	Effective dose range of 12.5, 25, and 50 mg/mL	[149]
Cervical, colon, and gastric cancer	HeLa, HT-29, AGS	ent-kauranoid glycosides (fructusnoids A-C) from extract of *X. strumarium* fruits	IC_50_ range between 7.6 μM to >50 μM	[150]
Breast cancer	MDA-MB-231, MCF-7	Ethanol extract of *Xylopia aethiopica* fruits	IC_50_ values of ~5 mg/mL	[180]
Cervical, oral, breast, and lung cancer	C-33A, KB, MCF-7, A549	Methanol extract of *X. aethiopica* fruits	IC_50_ values of 60.2 mg/mL (MCF-7), 62.5 mg/mL (KB), 30.8 mg/mL (C-33A), and >100 mg/mL (A549)	[179]
Leukemia, colon cancer	HCT116, U937, KG1a	Ent-15-oxokaur-16-en-19-oic acid isolated from ethanol extract of *X. aethiopica* fruits	IC_50_ values of 12 μg/mL (HCT116), 7.5 μg/mL (U937), and >25 μg/mL (KG1a)	[181]
Pancreatic cancer, leukemia	MiaPaCa-2, CCRF-CEM, multidrug-resistant CEM/ADR5000	Methanol extract of *X. aethiopica* fruits	IC_50_ values of 6.86 mg/mL (Mia PaCa2), 3.91 mg/mL (CCRF-CEM), and 7.4 mg/mL (CEM/ADR5000)	[182]
Leukemia, breast, liver and colon cancer, glioblastoma	Drug-sensitive CCRF-CEM and HL-60 cells, MDA-MB-231-pcDNA3, HCT116 (p53+/+), U87MG, HepG2 cells, multidrug-resistant HL-60/AR, MDA-MB-231-BCRP clone 23, HCT116 (p53−/−), U87MG.ΔEGFR	Methanol extracts of rhizomes of *E. giganteus*, roots of *l. cylindrica*, seeds of *P. capense* and *X. aethiopica*	IC_50_ values of 4.11 mg/mL (CCRF-CEM), 7.94 mg/mL (HL60), 30.60 mg/mL (HL60AR) 5.19 mg/mL (MDA-MB231) 10.04 mg/mL (MDA-MB231BCRP), 4.37 mg/mL (HCT116 p53+/+), and 18.28 mg/mL (HepG2)	[183]
Acute lymphoblastic leukemia, breast and colon cancer, glioblastoma	Drug-sensitive cell lines CCRF-CEM, MDA-MB-231-pcDNA3, HCT116 (p53+/+), U87MG and multidrug-resistant cell lines CEM/ADR5000, MDA-MB-231-BCRP clone 23, HCT116 (p53−/−), U87MG.ΔEGFR	Flavonoid 3,4′,5-trihydroxy-6”,6″-dimethylpyrano[2,3-γ] flavone, and alkaloid isotetrandrine derived from methanol crude extract of *X. aethiopica* fruits	IC_50_ range for flavonoid from 2.61 µM (CCRF-CEM) to 18.60 µM (U87MG.ΔEGFR) and alkaloid from 1.45 µM (HepG2) to 7.28 µM (MDA-MB-231-pcDNA)	[184]
Lung and gastric cancer	A549, AGS	Hydroethanol extracts of *X. aethiopica* fruits	Effective dose range of 500 mg/mL IC_50_ value for AGS of 151 mg/mL	[186]
Cervical and lung cancer	HeLa, MRC-5 SV2	olean-12-en-3-O-β-d-glucopyranoside, isoquercitrin and 5,7,3′,4′-tetrahydroxy-flavonol-3-O-[β-d-glucopyranosyl-(1→6)]-β-d-glucopyranoside derived from methanolic fruit extract of *Abelmoschus esculentus*	Relatively nontoxic or mildly toxic at a concentration of 100 mg/mL	[194]
Nonsmall cell lung cancer, breast, cervical, and liver cancer	NCL-H460, MCF-7, HeLa, HepG2	Extracts of *A. esculentus* pods	IC_50_ values of 49.62 mg/mL (NCL-H460), 56.40 mg/mL (MCF-7), 67.27 mg/mL (HeLa), and 167.95 mg/mL (HepG2)	[197]
Breast, liver, and cervical cancer	MCF-7, HepG2, HeLa	*A. esculentus* seed extract and ethanol, dichloromethane and petroleum fraction	Effective dose at 1000 μg/mL of seed extract	[198]
Breast cancer	MCF-7	*A. esculentus* lectin from *Abelmoschus esculentus*	Effective dose at 0.1 mg/mL	[200]
Glioblastoma	U87MG	*A. esculentus* lectin from Abelmoschus esculentus	IC_50_ 21 μg/mL	[201]
Breast, cervical, liver, and nonsmall cell lung cancer	MCF-7, HeLa, NCI-H460, HepG2	Hydroethanolic extract of *C. macrocarpa* fruits	IC_50_ values of 66 μg/mL (HeLa), 57 μg/mL (NCI-H460), 109 μg/mL (MCF-7), and >400 μg/mL (HepG2)	[119]
Breast, cervical, liver, lung, and brain cancer	MCF-7, HeLa, HEPG2, H460, U251	Alcoholic extract of *Syzygium cumini* fruits	IC_50_ values of 5.9 μg/mL (MCF-7) and >10 μg/mL (Hela, HEPG2, H460, and U251	[220]
Nonsmall lung cancer cells	A549	75% aqueous ethanol fruit pulp and seed powder of *S. cumini*	IC_50_ values of 59 μg/mL (pulp) 38 μg/mL (seeds)	[219]
Breast cancer	MCF-7aro, MDA-MB-231	Fruit pulp powder extract of *S. cumini*	IC_50_ values of 27 μg/mL (MCF-7aro) and 40 μg/mL (MDA-MB-231)	[221]
Cervical cancer	HeLa, SiHa	Crude and methanolic extract of *S. cumini* fruit skin	Crude extract inhibited 33.7% (HeLa) and 24.4% (SiHa) cell growth	[222]
Skin cancer	7,12-dimethyl benz(a)anthracene induced skin papilloma in vivo	Hydroalcoholic extract of *S. cumini* seeds	125 mg/kg/body wt./animal/day	[223]
Gastric cancer	Benzo[a]pyrene induced stomach carcinogenesis in vivo	Extract of *S. cumini* seeds	25 mg/kg body wt./day	[224]
Melanoma	G361	Dichloromethane extract and fractions from *Kigelia Africana* fruit	IC_50_ values of 2.1 mg/mL (extract), 1.2–15.4 mg/mL (fractions)	[240]
Melanoma	SK-MEL-28, MalMe-3M	Isolated phytocompounds from *K. Africana* fruit	IC_50_ value range of 0.3 to 180.5 mg/mL (SK-MEL-28), 0.5 to 75.5 mg/mL (MalMe-3M)	[241]
Colon cancer	Caco-2	n-Hexane extract of seed oil from *K. Africana* fruit	Effective dose of 20–120 mg/mL	[242]
Liver, pancreatic, colorectal, gastric, and colon cancer	Huh-7, PANC-1, Colo-205, HT-29, SNU-16, SW620, HCT116	Methanol and ethyl acetate extracts *K. Africana* fruit	IC_50_ range for methanolic extract from 6.79 µg/mL (SW620) to 91.32 µg/mL (HT-29) and ethyl acetate extract from 13.56 µg/mL (SNU-16) to 112.74 µg/mL (Huh-7)	[236]
Gastric cancer	Benzo[a]pyrene-induced stomach carcinogenesis in vivo	Ethanolic extract of *K. Africana* fruit	2 mg/day for eight weeks	[243]
Leukemia	Benzene-induced Leukemia in vivo	Ethanol extract *of K. Africana* fruit	0.5 mL of 100 mg/mL for three weeks	[244]
Prostate cancer	PC-3	Muricins M and N, and muricenin isolated from *Annona muricata* fruit extract	Effective concentration of 20 μg/mL	[255]
Liver cancer	HepG2, Hep-G2/2.2. 15	Muricin H, muricin I, and cis-annomontacin isolated from the seeds of *A. muricata*.	IC_50_ values for muricin H of 0.0951 μg/mL (HepG2), 0.01181 μg/mL (Hep-G2/2.2. 15), muricin I 0.0509 μg/mL (HepG2), 0.222 μg/mL (Hep-G2/2.2. 15), and cis-annomontacin 0.298 μg/mL (HepG2), and 0.0162 μg/mL (Hep-G2/2.2. 15)	[256]
Breast cancer	MDA-MB-468	*A. muricata* fruit extract	IC_50_ value of 4.8 μg/mL	[257]

## 4. Conclusions

Africa is home to a wide variety of medicinal plants, and South Africa contributes to approximately 20,000 different plant species, among which several have proved to be promising anticancer agents. Despite their traditional use, reports on plants used for the treatment of cancer are rare in the African continent. Among all of the plant parts investigated, fruits have been explored to a much lesser extent. Therefore, in this review, we tried to summarize the importance of several native or non-native fruits to Africa that have been screened against different cancer cell types. To the best of our knowledge, this study is the one of the first to provide information on the various fruits of different plant species native or cultivated in Africa, particularly in Southern Africa, with potential anticancer efficacy. Unfortunately, despite their health benefits, scientific reports on the investigation of the anticancer potentials of fruits from indigenous Southern African plant species are still unrecognized. Therefore, we widened our search results to include either native or widely cultivated plant species in Africa. The outcomes show that fruit extracts from *T. terrestris*, *X. strumarium*, *W. somnifera*, *X. aethiopica*, *A. esculentus*, *C. macrocarpa, C. edulis*, *S. cumini*, *K. Africana*, *A. muricata*, *P. americana*, *P. granatum*, *R. lancea*, *G. fruticosus*, and *A. digitata* have been investigated for their in vitro anticancer efficacy against several cancer types of cell lines. However, these studies are too limited and nonsystematic, which does not allow for reaching a decisive conclusion on the most effective fruit or fruit extract to extend to applications in preclinical and clinical studies.

Among all of the reported fruits, only the *Punica granatum* fruit has reached initial clinical studies for the treatment of prostate cancer. Many phytochemicals isolated from fruits have been investigated for their anticancer potential in clinical studies; however, as many studies suggest, an extract in combination with several different classes of phytochemicals can have better anticancer potency compared to isolated phytochemicals. Therefore, more thorough research is required to create safe and effective treatment plans that screen fruit extracts and identify potential new anticancer drugs. It is also essential to document and preserve crucial information regarding traditionally important fruits with anticancer effects as cultural heritage to serve as a foundation for scientific validation and the creation of more effective cancer therapeutics. More importantly, it is advised that all phytochemical substances be scientifically validated for their claimed efficacy, safety, and toxicity, because inadequate standardization, safety measures, quality control, and adulteration with conventional medicines pose a significant barrier to the use of phytochemicals and medicinal plant extracts in clinics. Investment in future research on the effects of African fruits in patients is needed as cost-effective and readily available treatment modalities/remedies because of the burden of cancer in Africa. Funding endeavors for natural products for cancer treatment would possibly assist in reducing this burden.

## Data Availability

Data sharing not applicable.

## References

[1] Sung H., Ferlay J., Siegel R.L., Laversanne M., Soerjomataram I., Jemal A., Bray F. (2021). Global Cancer Statistics 2020: GLOBOCAN Estimates of Incidence and Mortality Worldwide for 36 Cancers in 185 Countries. CA Cancer J. Clin..

[2] Sharma R., Aashima, Nanda M., Fronterre C., Sewagudde P., Ssentongo A.E., Yenney K., Arhin N.D., Oh J., Amponsah-Manu F. (2022). Mapping Cancer in Africa: A Comprehensive and Comparable Characterization of 34 Cancer Types Using Estimates From GLOBOCAN 2020. Front. Public Health.

[3] Bray F., Parkin D.M., Gnangnon F., Tshisimogo G., Peko J.-F., Adoubi I., Assefa M., Bojang L., Awuah B., Koulibaly M. (2022). Cancer in Sub-Saharan Africa in 2020: A Review of Current Estimates of the National Burden, Data Gaps, and Future Needs. Lancet Oncol..

[4] Bahnassy A.A., Abdellateif M.S., Zekri A.-R.N. (2020). Cancer in Africa: Is It a Genetic or Environmental Health Problem?. Front. Oncol..

[5] Cairncross L., Parkes J., Craig H., Are C. Cancer on the Global Stage: Incidence and Cancer-Related Mortality in South Africa—The ASCO Post. https://ascopost.com/issues/september-10-2021/cancer-on-the-global-stage-incidence-and-cancer-related-mortality-in-south-africa/.

[6] Lukong K.E., Ogunbolude Y., Kamdem J.P. (2017). Breast Cancer in Africa: Prevalence, Treatment Options, Herbal Medicines, and Socioeconomic Determinants. Breast Cancer Res. Treat..

[7] Hamdi Y., Abdeljaoued-Tej I., Zatchi A.A., Abdelhak S., Boubaker S., Brown J.S., Benkahla A. (2021). Cancer in Africa: The Untold Story. Front. Oncol..

[8] Jojo L.W., Nkutu N.T. (2023). Experiences of Patients on Cancer Treatment Regarding Decentralization of Oncology Services at a Tertiary Hospital in the Eastern Cape. BMC Cancer.

[9] Debela D.T., Muzazu S.G., Heraro K.D., Ndalama M.T., Mesele B.W., Haile D.C., Kitui S.K., Manyazewal T. (2021). New Approaches and Procedures for Cancer Treatment: Current Perspectives. SAGE Open Med..

[10] Baskar R., Lee K.A., Yeo R., Yeoh K.-W. (2012). Cancer and Radiation Therapy: Current Advances and Future Directions. Int. J. Med. Sci..

[11] Altun İ., Sonkaya A. (2018). The Most Common Side Effects Experienced by Patients Were Receiving First Cycle of Chemotherapy. Iran. J. Public Health.

[12] Remesh A. (2012). Toxicities of Anticancer Drugs and Its Management. Int. J. Basic Clin. Pharmacol..

[13] Brigden M., McKenzie M. (2000). Treating Cancer Patients. Practical Monitoring and Management of Therapy-Related Complications. Can. Fam. Physician Med. Fam. Can..

[14] Chu S.H., Lee Y.J., Lee E.S., Geng Y., Wang X.S., Cleeland C.S. (2015). Current Use of Drugs Affecting the Central Nervous System for Chemotherapy-Induced Peripheral Neuropathy in Cancer Patients: A Systematic Review. Support. Care Cancer Off. J. Multinatl. Assoc. Support. Care Cancer.

[15] Ewertz M., Qvortrup C., Eckhoff L. (2015). Chemotherapy-Induced Peripheral Neuropathy in Patients Treated with Taxanes and Platinum Derivatives. Acta Oncol. Stockh. Swed..

[16] Bidram E., Esmaeili Y., Ranji-Burachaloo H., Al-Zaubai N., Zarrabi A., Stewart A., Dunstan D.E. (2019). A Concise Review on Cancer Treatment Methods and Delivery Systems. J. Drug Deliv. Sci. Technol..

[17] Yuan H., Ma Q., Ye L., Piao G. (2016). The Traditional Medicine and Modern Medicine from Natural Products. Molecules.

[18] Choudhari A.S., Mandave P.C., Deshpande M., Ranjekar P., Prakash O. (2019). Phytochemicals in Cancer Treatment: From Preclinical Studies to Clinical Practice. Front. Pharmacol..

[19] Bouyahya A., Omari N.E., Bakrim S., Hachlafi N.E., Balahbib A., Wilairatana P., Mubarak M.S. (2022). Advances in Dietary Phenolic Compounds to Improve Chemosensitivity of Anticancer Drugs. Cancers.

[20] Elansary H.O., Szopa A., Kubica P., AAl-Mana F., Mahmoud E.A., Zin El-Abedin T.K.A., Mattar M.A., Ekiert H. (2019). Phenolic Compounds of Catalpa Speciosa, Taxus Cuspidata, and Magnolia Acuminata Have Antioxidant and Anticancer Activity. Molecules.

[21] Dobrzynska M., Napierala M., Florek E. (2020). Flavonoid Nanoparticles: A Promising Approach for Cancer Therapy. Biomolecules.

[22] ul Islam B., Suhail M., Khan M.S., Ahmad A., Zughaibi T.A., Husain F.M., Rehman M.T., Tabrez S. (2021). Flavonoids and PI3K/Akt/MTOR Signaling Cascade: A Potential Crosstalk in Anticancer Treatment. Curr. Med. Chem..

[23] Moyo M., Aremu A.O., Van Staden J. (2015). Medicinal Plants: An Invaluable, Dwindling Resource in Sub-Saharan Africa. J. Ethnopharmacol..

[24] The Use of African Medicinal Plants in Cancer Management—PMC. https://www.ncbi.nlm.nih.gov/pmc/articles/PMC9971233/.

[25] Tsoka-Gwegwenia J.M., Cumber S.N., Nchanji K.N. (2017). Breast Cancer among Women in Sub-Saharan Africa: Prevalence and a Situational Analysis. S. Afr. J. Gynaecol. Oncol..

[26] Ma J., Jemal A., Ahmad A. (2013). Breast Cancer Statistics. Breast Cancer Metastasis and Drug Resistance: Progress and Prospects.

[27] Thomford N.E., Senthebane D.A., Rowe A., Munro D., Seele P., Maroyi A., Dzobo K. (2018). Natural Products for Drug Discovery in the 21st Century: Innovations for Novel Drug Discovery. Int. J. Mol. Sci..

[28] Daga A., Ansari A., Patel S., Mirza S., Rawal R., Umrania V. (2015). Current Drugs and Drug Targets in Non-Small Cell Lung Cancer: Limitations and Opportunities. Asian Pac. J. Cancer Prev. APJCP.

[29] Ashraf M.A. (2020). Phytochemicals as Potential Anticancer Drugs: Time to Ponder Nature’s Bounty. BioMed Res. Int..

[30] Egbuna C., Kumar S., Ifemeje J.C., Ezzat S.M., Kaliyaperumal S. (2019). Phytochemicals as Lead Compounds for New Drug Discovery.

[31] Berdigaliyev N., Aljofan M. (2020). An Overview of Drug Discovery and Development. Future Med. Chem..

[32] Shankar M.G., Swetha M., Keerthana C.K., Rayginia T.P., Anto R.J. (2021). Cancer Chemoprevention: A Strategic Approach Using Phytochemicals. Front. Pharmacol..

[33] Newman D.J., Cragg G.M. (2020). Natural Products as Sources of New Drugs over the Nearly Four Decades from 01/1981 to 09/2019. J. Nat. Prod..

[34] Twilley D., Rademan S., Lall N. (2020). A Review on Traditionally Used South African Medicinal Plants, Their Secondary Metabolites and Their Potential Development into Anticancer Agents. J. Ethnopharmacol..

[35] Fouche G., Cragg G.M., Pillay P., Kolesnikova N., Maharaj V.J., Senabe J. (2008). In Vitro Anticancer Screening of South African Plants. J. Ethnopharmacol..

[36] Mokganya M.G., Tshisikhawe M.P. (2019). Medicinal Uses of Selected Wild Edible Vegetables Consumed by Vhavenda of the Vhembe District Municipality, South Africa. S. Afr. J. Bot..

[37] Pfukwa T.M., Chikwanha O.C., Katiyatiya C.L.F., Fawole O.A., Manley M., Mapiye C. (2020). Southern African Indigenous Fruits and Their Byproducts: Prospects as Food Antioxidants. J. Funct. Foods.

[38] Pfukwa T.M., Fawole O.A., Manley M., Mapiye C. (2022). Phenolic Profiling and Antioxidant Evaluation of Extracts from Southern African Indigenous Fruits Byproducts. Food Res. Int..

[39] Aqil F., Munagala R., Jeyabalan J., Joshi T., Gupta R.C., Singh I.P., Preedy V. (2014). Chapter 10—The Indian Blackberry (Jamun), Antioxidant Capacity, and Cancer Protection. Cancer.

[40] Kamanula M., Munthali C.Y., Kamanula J.F. (2022). Nutritional and Phytochemical Variation of Marula (*Sclerocarya birrea*) (Subspecies *Caffra* and *Birrea*) Fruit among Nine International Provenances Tested in Malawi. Int. J. Food Sci..

[41] Nitcheu Ngemakwe P.H., Remize F., Thaoge M.L., Sivakumar D. (2017). Phytochemical and Nutritional Properties of Underutilised Fruits in the Southern African Region. S. Afr. J. Bot..

[42] Mashau M.E., Kgatla T.E., Makhado M.V., Mikasi M.S., Ramashia S.E. (2022). Nutritional Composition, Polyphenolic Compounds and Biological Activities of Marula Fruit (*Sclerocarya birrea*) with Its Potential Food Applications: A Review. Int. J. Food Prop..

[43] Ojewole J.A.O., Mawoza T., Chiwororo W.D.H., Owira P.M.O. (2010). *Sclerocarya birrea* (a. Rich) Hochst. [‘Marula’] (Anacardiaceae): A Review of Its Phytochemistry, Pharmacology and Toxicology and Its Ethnomedicinal Uses. Phytother. Res..

[44] Prinsloo G., Street R.A. (2013). Marula [*Sclerocarya birrea* (A.Rich) Hochst]: A Review of Traditional Uses, Phytochemistry, and Pharmacology. African Natural Plant Products Volume II: Discoveries and Challenges in Chemistry, Health, and Nutrition.

[45] Mariod A.A., Abdelwahab S.I. (2012). *Sclerocarya birrea* (Marula), An African Tree of Nutritional and Medicinal Uses: A Review. Food Rev. Int..

[46] Eloff J.N. (2001). Antibacterial Activity of Marula (*Sclerocarya birrea* (A. Rich.) Hochst. Subsp. Caffra (Sond.) Kokwaro) (Anacardiaceae) Bark and Leaves. J. Ethnopharmacol..

[47] Ojewole J.A.O. (2003). Evaluation of the Anti-Inflammatory Properties of *Sclerocarya birrea* (A. Rich.) Hochst. (Family: Anacardiaceae) Stem-Bark Extracts in Rats. J. Ethnopharmacol..

[48] Muhammad S., Hassan L.G., Dangoggo S.M., Hassan S.W., Umar R.A., Umar K.J. (2014). Acute and Subchronic Toxicity Studies of *Sclerocarya birrea* Peels Extract in Rats. Int. J. Sci. Basic Appl. Res. IJSBAR.

[49] McGaw L.J., Van der Merwe D., Eloff J.N. (2007). In Vitro Anthelmintic, Antibacterial and Cytotoxic Effects of Extracts from Plants Used in South African Ethnoveterinary Medicine. Vet. J..

[50] Gathirwa J.W., Rukunga G.M., Njagi E.N.M., Omar S.A., Mwitari P.G., Guantai A.N., Tolo F.M., Kimani C.W., Muthaura C.N., Kirira P.G. (2008). The in Vitro Anti-Plasmodial and in Vivo Anti-Malarial Efficacy of Combinations of Some Medicinal Plants Used Traditionally for Treatment of Malaria by the Meru Community in Kenya. J. Ethnopharmacol..

[51] Gondwe M., Kamadyaapa D.R., Tufts M., Chuturgoon A.A., Musabayane C.T. (2008). *Sclerocarya birrea* [(A. Rich.) Hochst.] [Anacardiaceae] Stem-Bark Ethanolic Extract (SBE) Modulates Blood Glucose, Glomerular Filtration Rate (GFR) and Mean Arterial Blood Pressure (MAP) of STZ-Induced Diabetic Rats. Phytomedicine.

[52] Borochov-Neori H., Judeinstein S., Greenberg A., Fuhrman B., Attias J., Volkova N., Hayek T., Aviram M. (2008). Phenolic Antioxidants and Antiatherogenic Effects of Marula (*Sclerocarrya birrea* Subsp. Caffra) Fruit Juice in Healthy Humans. J. Agric. Food Chem..

[53] Mpai S., du Preez R., Sultanbawa Y., Sivakumar D. (2018). Phytochemicals and Nutritional Composition in Accessions of Kei-Apple (*Dovyalis caffra*): Southern African Indigenous Fruit. Food Chem..

[54] du Preez R.J., Caballero B. (2003). Fruits of Tropical Climates | Lesser-Known Fruits of Africa. Encyclopedia of Food Sciences and Nutrition.

[55] Aremu A.O., Ncama K., Omotayo A.O. (2019). Ethnobotanical Uses, Biological Activities and Chemical Properties of Kei-Apple [*Dovyalis caffra* (Hook.f. & Harv.) Sim]: An Indigenous Fruit Tree of Southern Africa. J. Ethnopharmacol..

[56] Omotayo A.O., Aremu A.O. (2020). Underutilized African Indigenous Fruit Trees and Food–Nutrition Security: Opportunities, Challenges, and Prospects. Food Energy Secur..

[57] Qanash H., Yahya R., Bakri M.M., Bazaid A.S., Qanash S., Shater A.F., T.M. A. (2022). Anticancer, Antioxidant, Antiviral and Antimicrobial Activities of Kei Apple (*Dovyalis caffra*) Fruit. Sci. Rep..

[58] El-Menshawi B.S., Fayad W., Mahmoud K., El-Hallouty S.M., El-Manawaty M., Olofsson M.H., Linder S. (2010). Screening of Natural Products for Therapeutic Activity against Solid Tumors. Indian J. Exp. Biol..

[59] Moustafa S., Menshawi B.M., Wassel G., Mahmoud K., Mounier M.M. (2014). Screening of Some Plants in Egypt for Their Cytotoxicity against Four Human Cancer Cell Lines. Int. J. PharmTech Res..

[60] Taher M.A., Tadros L.K., Dawood D.H. (2018). Phytochemical Constituents, Antioxidant Activity and Safety Evaluation of Kei-Apple Fruit (*Dovyalis caffra*). Food Chem..

[61] Akweni A.L., Sibanda S., Zharare G.E., Zimudzi C. (2020). Fruit-Based Allometry of *Strychnos madagascariensis* and *S. Spinosa* (Loganiaceae) in the Savannah Woodlands of the Umhlabuyalingana Municipality, KwaZulu-Natal, South Africa. Trees For. People.

[62] Ogbonnia S., Preedy V.R., Watson R.R., Patel V.B. (2011). Chapter 91—Extracts of Mobola Plum (*Parinari curatellifolia* Planch Ex Benth, Chrysobalanaceae) Seeds and Multiple Therapeutic Activities. Nuts and Seeds in Health and Disease Prevention.

[63] *Parinari curatellifolia*|PlantZAfrica. https://pza.sanbi.org/parinari-curatellifolia.

[64] Crown O.O., Olayeriju O.S., Kolawole A.O., Akinmoladun A.C., Olaleye M.T., Akindahunsi A.A. (2017). Mobola Plum Seed Methanolic Extracts Exhibit Mixed Type Inhibition of Angiotensin Ⅰ-Converting Enzyme in Vitro. Asian Pac. J. Trop. Biomed..

[65] Olaleye M.T., Amobonye A.E., Komolafe K., Akinmoladun A.C. (2014). Protective Effects of *Parinari curatellifolia* Flavonoids against Acetaminophen-Induced Hepatic Necrosis in Rats. Saudi J. Biol. Sci..

[66] Manuwa T.R., Akinmoladun A.C., Crown O.O., Komolafe K., Olaleye M.T. (2017). Toxicological Assessment and Ameliorative Effects of *Parinari curatellifolia* Alkaloids on Triton-Induced Hyperlipidemia and Atherogenicity in Rats. Proc. Natl. Acad. Sci. India Sect. B Biol. Sci..

[67] Nkosi N.J., Shoko T., Manhivi V.E., Slabbert R.M., Sultanbawa Y., Sivakumar D. (2022). Metabolomic and Chemometric Profiles of Ten Southern African Indigenous Fruits. Food Chem..

[68] Studies of In Vitro Antioxidant and Cytotoxic Activities of Extracts and Isolated Compounds from Parinari curatellifolia (Chrysobalanaceae)|Semantic Scholar. https://www.semanticscholar.org/paper/Studies-of-In-vitro-Antioxidant-and-Cytotoxic-of-Halilu-October/d853e8e182761bb56d884215ed7eed96e6972433.

[69] Ogbonnia S.O., Olayemi S.O., Anyika E.N., Enwuru V.N., Poluyi O.O. (2009). Evaluation of Acute Toxicity in Mice and Subchronic Toxicity of Hydroethanolic Extract of *Parinari curatellifolia* Planch (Chrysobalanaceae) Seeds in Rats. Afr. J. Biotechnol..

[70] Halilu E.M., October N., Ugwah-Oguejiofor C.J., Jega A.Y., Nefai M.S. (2020). Anti-Snake Venom and Analgesic Activities of Extracts and Betulinic and Oleanolic Acids Isolated from *Parinari curatellifolia*. J. Med. Plants Econ. Dev..

[71] Halilu E. (2023). Toxicity Assessment of Methanol Extract of *Parinari curatellifolia* Planch Ex. Benth. Pak. J. Pharm. Sci..

[72] Mngadi S., Moodley R., Jonnalagadda S.B. (2017). Elemental Composition and Nutritional Value of the Edible Fruits of Coastal Red-Milkwood (*Mimusops caffra*) and Impact of Soil Quality on Their Chemical Characteristics. J. Environ. Sci. Health Part B.

[73] Mashela P.W., Pofu K.M., Nzanza B. (2013). Responses of Mmupudu (*Mimusops zeyheri*) Indigenous Fruit Tree to Three Soil Types. Afr. J. Agric. Res..

[74] Chivandi E., Davidson B., Pretorius B., Erlwanger K. (2011). Proximate, Mineral, Amino Acid, Fatty Acid, Vitamin E, Phytate Phosphate and Fibre Composition of *Mimusops zeyheri* (Red Milkwood) Seed. Int. J. Food Sci. Technol..

[75] Chivandi E., Mukonowenzou N., Berliner D. (2016). The Coastal Red-Milkwood (*Mimusops caffra*) Seed: Proximate, Mineral, Amino Acid and Fatty Acid Composition. S. Afr. J. Bot..

[76] Omotayo A.O., Ijatuyi E.J., Ogunniyi A.I., Aremu A.O. (2020). Exploring the Resource Value of Transvaal Red Milk Wood (*Mimusops zeyheri*) for Food Security and Sustainability: An Appraisal of Existing Evidence. Plants.

[77] De Wet H., Nzama V.N., Van Vuuren S.F. (2012). Medicinal Plants Used for the Treatment of Sexually Transmitted Infections by Lay People in Northern Maputaland, KwaZulu–Natal Province, South Africa. S. Afr. J. Bot..

[78] Neuwinger H.D. (2000). African Traditional Medicine: A Dictionary of Plant Use and Applications. With Supplement: Search System for Diseases.

[79] Madiehe A.M., Moabelo K.L., Modise K., Sibuyi N.R., Meyer S., Dube A., Onani M.O., Meyer M. (2022). Catalytic Reduction of 4-Nitrophenol and Methylene Blue by Biogenic Gold Nanoparticles Synthesized Using *Carpobrotus edulis* Fruit (Sour Fig) Extract. Nanomater. Nanotechnol..

[80] Hafsa J., Hammi K.M., Khedher M.R.B., Smach M.A., Charfeddine B., Limem K., Majdoub H. (2016). Inhibition of Protein Glycation, Antioxidant and Antiproliferative Activities of *Carpobrotus edulis* Extracts. Biomed. Pharmacother..

[81] Malan C., Notten A. Carpobrotus Edulis|PlantZAfrica. http://pza.sanbi.org/carpobrotus-edulis.

[82] Omoruyi B.E., Bradley G., Afolayan A.J. (2012). Antioxidant and Phytochemical Properties of *Carpobrotus edulis* (L.) Bolus Leaf Used for the Management of Common Infections in HIV/AIDS Patients in Eastern Cape Province. BMC Complement. Altern. Med..

[83] Otang-Mbeng F.N.N., Simeon A. (2019). Materechera, Wilfred *Carpobrotus edulis* L. (Sour Fig): Phytochemistry, Pharmacology, and Toxicology. The Therapeutic Properties of Medicinal Plants.

[84] Martins A., Vasas A., Viveiros M., Molnár J., Hohmann J., Amaral L. (2011). Antibacterial Properties of Compounds Isolated from *Carpobrotus edulis*. Int. J. Antimicrob. Agents.

[85] Omoruyi B.E., Afolayan A.J., Bradley G. (2014). Chemical Composition Profiling and Antifungal Activity of the Essential Oil and Plant Extracts of *Mesembryanthemum edule* (L.) Bolus Leaves. Afr. J. Tradit. Complement. Altern. Med..

[86] Omoruyi S.I., Enogieru A.B., Ekpo O.E. (2021). In Vitro Evaluation of the Antiproliferative Activity of *Carpobrotus edulis* on Human Neuroblastoma Cells. J. Herb. Med..

[87] Ordway D., Hohmann J., Viveiros M., Viveiros A., Molnar J., Leandro C., Arroz M.J., Gracio M.A., Amaral L. (2003). *Carpobrotus edulis* Methanol Extract Inhibits the MDR Efflux Pumps, Enhances Killing of Phagocytosed *S. Aureus* and Promotes Immune Modulation. Phytother. Res..

[88] Maroyi A. (2018). Nutraceutical and Ethnopharmacological Properties of *Vangueria infausta* Subsp. Infausta. Molecules.

[89] Achilonu M.C., Ngubane X.V., Nkosi S.M., Jiyane P.C. (2023). Phytochemicals, Bioactivity, and Ethnopharmacological Potential of Selected Indigenous Plants. S. Afr. J. Sci..

[90] Vangueria Esculenta|PlantZAfrica. https://pza.sanbi.org/vangueria-esculenta.

[91] Raice R.T., Sjoholm I., Wang H., Bergenståhl B. (2015). Characterization of Volatile Components Extracted from *Vangueria infausta* (African Medlar) by Using GC–MS. J. Essent. Oil Res..

[92] Tshikalange T.E., Mophuting B.C., Mahore J., Winterboer S., Lall N. (2016). An Ethnobotanical Study of Medicinal Plants Used in Villages under Jongilanga Tribal Council, Mpumalanga, South Africa. Afr. J. Tradit. Complement. Altern. Med..

[93] Antibacterial Activity of Sixteen Plant Species from Phalaborwa, Limpopo Province, South Africa | Semantic Scholar. https://www.semanticscholar.org/paper/Antibacterial-activity-of-sixteen-plant-species-Shai-Chauke/ab9b7c34b448c3828f74136a9524aba7a214b3fc.

[94] Munodawafa T., Chagonda L.S., Moyo S.R. (2013). Antimicrobial and Phytochemical Screening of Some Zimbabwean Medicinal Plants. J. Biol. Act. Prod. Nat..

[95] Mbukwa E., Chacha M., Majinda R.R.T. (2006). Phytochemical Constituents of *Vangueria infausta*: Their Radical Scavenging and Antimicrobial Activities. Arkivoc.

[96] Mmushi T., Masoko P., Mdee L., Mokgotho M., Mampuru L., Howard R. (2009). Antimycobacterial Evaluation of Fifteen Medicinal Plants in South Africa. Afr. J. Tradit. Complement. Altern. Med..

[97] Mahlo S.M., Chauke H.R., McGaw L., Eloff J. (2016). Antioxidant and Antifungal Activity of Selected Medicinal Plant Extracts against Phytopathogenic Fungi. Afr. J. Tradit. Complement. Altern. Med..

[98] Bapela M.J., Kaiser M., Meyer J.J.M. (2017). Antileishmanial Activity of Selected South African Plant Species. S. Afr. J. Bot..

[99] Abosl A.O., Mbukwa E., Majinda R.R., Raserok B.H., Yenesew A., Midiwo J.O., Akala H., Liyala P., Waters N.C. (2006). *Vangueria infausta* Root Bark: In Vivo and in Vitro Antiplasmodial Activity. Br. J. Biomed. Sci..

[100] Bapela M.J., Heyman H., Senejoux F., Meyer J.J.M. (2019). ^1^H NMR-Based Metabolomics of Antimalarial Plant Species Traditionally Used by Vha-Venda People in Limpopo Province, South Africa and Isolation of Antiplasmodial Compounds. J. Ethnopharmacol..

[101] Morobe I.C., Mthethwa N.S., Bisi-Johnson M.A., Vasaikar S.D., Obi C.L., Oyedeji A.O., Kambizi L., Eloff J.N., Hattori T. (2012). Cytotoxic Effects and Safety Profiles of Extracts of Active Medicinal Plants from South Africa. J. Microbiol. Res..

[102] Bapela M.J., Meyer J.J.M., Kaiser M. (2014). In Vitro Antiplasmodial Screening of Ethnopharmacologically Selected South African Plant Species Used for the Treatment of Malaria. J. Ethnopharmacol..

[103] Aro A.O., Dzoyem J.P., Hlokwe T.M., Madoroba E., Eloff J.N., McGaw L.J. (2015). Some South African Rubiaceae Tree Leaf Extracts Have Antimycobacterial Activity Against Pathogenic and Non-Pathogenic Mycobacterium Species. Phytother. Res..

[104] Moshi M.J., Innocent E., Magadula J.J., Otieno D.F., Weisheit A., Mbabazi P.K., Nondo R.S.O. (2010). Brine Shrimp Toxicity of Some Plants Used as Traditional Medicines in Kagera Region, North Western Tanzania. Tanzan. J. Health Res..

[105] Nawwar M., Hussein S., Ayoub N., Hashim A., El-Sharawy R., Lindequist U., Harms M., Wende K. (2011). Constitutive Phenolics of *Harpephyllum caffrum* (Anacardiaceae) and Their Biological Effects on Human Keratinocytes. Fitoterapia.

[106] Olofinsan K.A., Salau V.F., Erukainure O.L., Islam M.S. (2022). *Harpephyllum caffrum* Fruit (Wild Plum) Facilitates Glucose Uptake and Modulates Metabolic Activities Linked to Neurodegeneration in Isolated Rat Brain: An in Vitro and in Silico Approach. J. Food Biochem..

[107] Moodley R., Koorbanally N., Jonnalagadda S.B. (2013). Elemental Composition and Nutritional Value of the Edible Fruits of *Harpephyllum caffrum* and Impact of Soil Quality on Their Chemical Characteristics. J. Environ. Sci. Health Part B.

[108] Harpephyllum Caffrum|PlantZAfrica. https://pza.sanbi.org/harpephyllum-caffrum.

[109] Shabana M.M., El Sayed A.M., Yousif M.F., El Sayed A.M., Sleem A.A. (2011). Bioactive Constituents from *Harpephyllum caffrum* Bernh. and *Rhus coriaria* L.. Pharmacogn. Mag..

[110] Mapunya M.B., Nikolova R.V., Lall N. (2012). Melanogenesis and Antityrosinase Activity of Selected South African Plants. Evid. Based Complement. Alternat. Med..

[111] Khalil H.E., Aljeshi Y.M., Saleh F.A. (2015). Authentication of *Carissa macrocarpa* Cultivated in Saudi Arabia; Botanical, Phytochemical and Genetic Study. J. Pharm. Sci. Res..

[112] Mphaphuli T., Slabbert R.M., Sivakumar D. (2020). Storage Temperature and Time Changes of Phenolic Compounds and Antioxidant Properties of Natal Plum (*Carissa macrocarpa*). Food Biosci..

[113] Souilem F., Dias M.I., Barros L., Calhelha R.C., Alves M.J., Harzallah-Skhiri F., Ferreira I.C.F.R. (2019). Phenolic Profile and Bioactive Properties of *Carissa macrocarpa* (Eckl.) A.DC.: An In Vitro Comparative Study between Leaves, Stems, and Flowers. Molecules.

[114] Seke F., Manhivi V.E., Slabbert R.M., Sultanbawa Y., Sivakumar D. (2022). In Vitro Release of Anthocyanins from Microencapsulated Natal Plum (*Carissa macrocarpa*) Phenolic Extract in Alginate/Psyllium Mucilage Beads. Foods.

[115] Venter F., Venter J.A., Joffe P. (2016). Making the Most of Indigenous Trees.

[116] Food, Pharmaceutical and Industrial Potential of Carissa Genus: An Overview|SpringerLink. https://link.springer.com/article/10.1007/s11157-012-9306-7.

[117] Kaunda J.S., Zhang Y.-J. (2017). The Genus Carissa: An Ethnopharmacological, Phytochemical and Pharmacological Review. Nat. Prod. Bioprospecting.

[118] Moodley R., Chenia H., Jonnalagadda S.B., Koorbanally N. (2011). Antibacterial and Anti-Adhesion Activity of the Pentacyclic Triterpenoids Isolated from the Leaves and Edible Fruits of *Carissa macrocarpa*. J. Med. Plants Res..

[119] Souilem F., Dias M.I., Barros L., Calhelha R.C., Alves M.J., Harzallah-Skhiri F., Ferreira I.C.F.R. (2019). Amantagula Fruit (*Carissa macrocarpa* (Eckl.) A.DC.): Nutritional and Phytochemical Characterization. Plant Foods Hum. Nutr..

[120] Harpephyllum Caffrum—Useful Tropical Plants. https://tropical.theferns.info/viewtropical.php?id=Harpephyllum+caffrum.

[121] Sivakumar D., Remize F., Garcia C., Murthy H.N., Bapat V.A. (2019). Bioactive Compounds in Southern African Fruits. Bioactive Compounds in Underutilized Fruits and Nuts.

[122] Moodley R. Phytochemical and Analytical Studies on Two Indigenous Medicinal Plants Found in KwaZulu-Natal, South Africa; *Carissa macrocarpa* and *Harpephyllum caffrum*. Ph.D. Thesis.

[123] Tkachenko K., Frontasyeva M., Vasilev A., Avramov L., Shi L. (2020). Major and Trace Element Content of *Tribulus terrestris* L. Wildlife Plants. Plants.

[124] Zhu W., Du Y., Meng H., Dong Y., Li L. (2017). A Review of Traditional Pharmacological Uses, Phytochemistry, and Pharmacological Activities of *Tribulus terrestris*. Chem. Cent. J..

[125] Chhatre S., Nesari T., Somani G., Kanchan D., Sathaye S. (2014). Phytopharmacological Overview of *Tribulus terrestris*. Pharmacogn. Rev..

[126] Semerdjieva I.B., Zheljazkov V.D. (2019). Chemical Constituents, Biological Properties, and Uses of *Tribulus terrestris*: A Review. Nat. Prod. Commun..

[127] Yanala S.R., Sathyanarayana D., Kannan K. (2016). A Recent Phytochemical Review—Fruits of *Tribulus terrestris* Linn. J Pharm Sci.

[128] Yan W., Ohtani K., Kasai R., Yamasaki K. (1996). Steroidal Saponins from Fruits of *Tribulus terrestris*. Phytochemistry.

[129] Chang H.-W., Liu P.-F., Tsai W.-L., Hu W.-H., Hu Y.-C., Yang H.-C., Lin W.-Y., Weng J.-R., Shu C.-W. (2019). *Xanthium strumarium* Fruit Extract Inhibits ATG4B and Diminishes the Proliferation and Metastatic Characteristics of Colorectal Cancer Cells. Toxins.

[130] Shu C.-W., Weng J.-R., Chang H.-W., Liu P.-F., Chen J.-J., Peng C.-C., Huang J.-W., Lin W.-Y., Yen C.-Y. (2021). *Tribulus terrestris* Fruit Extract Inhibits Autophagic Flux to Diminish Cell Proliferation and Metastatic Characteristics of Oral Cancer Cells. Environ. Toxicol..

[131] Kim H.J., Kim J.C., Min J.S., Kim M., Kim J.A., Kor M.H., Yoo H.S., Ahn J.K. (2011). Aqueous Extract of *Tribulus terrestris* Linn Induces Cell Growth Arrest and Apoptosis by Down-Regulating NF-ΚB Signaling in Liver Cancer Cells. J. Ethnopharmacol..

[132] Kumar M., Soni A.K., Shukla S., Kumar A. (2006). Chemopreventive Potential of *Tribulus terrestris* against 7,12- Dimethylbenz (a) Anthracene Induced Skin Papillomagenesis in Mice. Asian Pac. J. Cancer Prev..

[133] Hanif M.A., Yousaf S., Rehman R., Hanif A., Nadeem R., Hanif M.A., Nawaz H., Khan M.M., Byrne H.J. (2020). Chapter 42—Puncture Vine. Medicinal Plants of South Asia.

[134] Ștefănescu R., Tero-Vescan A., Negroiu A., Aurică E., Vari C.-E. (2020). A Comprehensive Review of the Phytochemical, Pharmacological, and Toxicological Properties of *Tribulus terrestris* L.. Biomolecules.

[135] El-Shaibany A., AL-Habori M., Al-Tahami B., Massarani S.A. (2015). Anti-Hyperglycaemic Activity of *Tribulus terrestris* L Aerial Part Extract in Glucose-Loaded Normal Rabbits. Trop. J. Pharm. Res..

[136] Hemalatha S., Hari R. (2014). Acute and Subacute Toxicity Studies of the Saponin Rich Butanol Extracts of *Tribulus terrestris* Fruits in Wistar Rats. Int. J. Pharm. Sci. Rev. Res..

[137] Gandhi S., Srinivasan B.P., Akarte A.S. (2013). Potential Nephrotoxic Effects Produced by Steroidal Saponins from Hydro Alcoholic Extract of *Tribulus terrestris* in STZ-Induced Diabetic Rats. Toxicol. Mech. Methods.

[138] Aslani M.R., Movassaghi A.R., Mohri M., Pedram M., Abavisani A. (2003). Experimental *Tribulus terrestris* Poisoning in Sheep: Clinical, Laboratory and Pathological Findings. Vet. Res. Commun..

[139] Abudayyak M., Jannuzzi A.T., Özhan G., Alpertunga B. (2015). Investigation on the Toxic Potential of *Tribulus terrestris* in Vitro. Pharm. Biol..

[140] Talasaz A.H., Abbasi M.-R., Abkhiz S., Dashti-Khavidaki S. (2010). *Tribulus terrestris*-Induced Severe Nephrotoxicity in a Young Healthy Male. Nephrol. Dial. Transplant. Off. Publ. Eur. Dial. Transpl. Assoc.—Eur. Ren. Assoc..

[141] Campanelli M., De Thomasis R., Tenaglia R.L. (2016). Priapism Caused by “*Tribulus terrestris*”. Int. J. Impot. Res..

[142] Ryan M., Lazar I., Nadasdy G.M., Nadasdy T., Satoskar A.A. (2015). Acute Kidney Injury and Hyperbilirubinemia in a Young Male after Ingestion of *Tribulus terrestris*. Clin. Nephrol..

[143] Factsheet—Xanthium strumarium (Large Cocklebur). https://keys.lucidcentral.org/keys/v3/eafrinet/weeds/key/weeds/Media/Html/Xanthium_strumarium_(Large_Cocklebur).htm.

[144] Fan W., Fan L., Peng C., Zhang Q., Wang L., Li L., Wang J., Zhang D., Peng W., Wu C. (2019). Traditional Uses, Botany, Phytochemistry, Pharmacology, Pharmacokinetics and Toxicology of *Xanthium strumarium* L.: A Review. Molecules.

[145] Kamboj A., Saluja A.K. (2010). Phytopharmacological Review of *Xanthium strumarium* L. (Cocklebur). Int. J. Green Pharm. IJGP.

[146] Al-Mekhlafi F.A., Abutaha N., Mashaly A.M.A., Nasr F.A., Ibrahim K.E., Wadaan M.A. (2017). Biological Activity of *Xanthium strumarium* Seed Extracts on Different Cancer Cell Lines and *Aedes Caspius*, *Culex Pipiens* (Diptera: Culicidae). Saudi J. Biol. Sci..

[147] Xu X.-W., Xi Y.-Y., Chen J., Zhang F., Zheng J.-J., Zhang P.-H. (2022). Phytochemical Investigation of the Fruits of *Xanthium strumarium* and Their Cytotoxic Activity. J. Nat. Med..

[148] Didem Karagoz I., Cakir A., Ozaslan M., Halil Kilic I., Tepe B., Akdogan E., Kazaz C. (2022). Anticancer Agents from *Xanthium strumarium* Fruits Against C6 Glioma Cells. Int. J. Pharmacol..

[149] Vaishnav K., George L.-B., Highland H.N. (2015). Induction of Cell Death through Alteration of Antioxidant Activity in HeLa Cervical Cancer Cells by *Xanthium strumarium* L. Extract. IOSR J. Pharm. Biol. Sci..

[150] Jiang H., Ma G.-X., Yang L., Xing X.-D., Yan M.-L., Zhang Y.-Y., Wang Q.-H., Kuang H.-X., Xu X.-D. (2016). Rearranged Ent-Kauranoid Glycosides from the Fruits of *Xanthium strumarium* and Their Antiproliferative Activity. Phytochem. Lett..

[151] Stuart B.P., Cole R.J., Gosser H.S. (1981). Cocklebur (*Xanthium strumarium*, L. Var. *Strumarium*) Intoxication in Swine: Review and Redefinition of the Toxic Principle. Vet. Pathol..

[152] Yan L., Zhang T., Zhao J., Song J., Hua H., Li L. (2012). [Comparative study on acute toxicity of four extracts from Xanthii Fructus in mice]. Zhongguo Zhong Yao Za Zhi Zhongguo Zhongyao Zazhi China J. Chin. Mater. Medica.

[153] Jin Y., Liu S., Liu Y., Mou H. (2010). Toxic Effects of Ethyl Acetate, n-Butanol, and Water Extracts from Alcohol Extractions of Cocklebur Fruit on Liver in Rats. Adverse Drug React. J..

[154] Wang Y., Han T., Xue L.-M., Han P., Zhang Q.-Y., Huang B.-K., Zhang H., Ming Q.-L., Peng W., Qin L.-P. (2011). Hepatotoxicity of Kaurene Glycosides from *Xanthium strumarium* L. Fruits in Mice. Pharm..

[155] Xue L.-M., Zhang Q.-Y., Han P., Jiang Y.-P., Yan R.-D., Wang Y., Rahman K., Jia M., Han T., Qin L.-P. (2014). Hepatotoxic Constituents and Toxicological Mechanism of *Xanthium strumarium* L. Fruits. J. Ethnopharmacol..

[156] Mandal S.C., Dhara A.K., Ashok Kumar C.K., Maiti B.C. (2001). Neuropharmacological Activity of *Xanthium strumarium* Linn. Extract. J. Herbs Spices Med. Plants.

[157] Xanthium strumarium L. Extracts Produce DNA Damage Mediated by Cytotoxicity in in Vitro Assays but Does Not Induce Micronucleus in Mice—PubMed. https://pubmed.ncbi.nlm.nih.gov/25025061/.

[158] Saidi H., Mofidi M. (2009). Toxic Effect of *Xanthium strumarium* as an Herbal Medicine Preparation. EXCLI J..

[159] Oza V.P., Parmar P.P., Kumar S., Subramanian R.B. (2010). Anticancer Properties of Highly Purified L-Asparaginase from *Withania somnifera* L. against Acute Lymphoblastic Leukemia. Appl. Biochem. Biotechnol..

[160] Afewerky H.K., Ayodeji A.E., Tiamiyu B.B., Orege J.I., Okeke E.S., Oyejobi A.O., Bate P.N.N., Adeyemi S.B. (2021). Critical Review of the *Withania somnifera* (L.) Dunal: Ethnobotany, Pharmacological Efficacy, and Commercialization Significance in Africa. Bull. Natl. Res. Cent..

[161] Withania Somnifera|PlantZAfrica. http://pza.sanbi.org/withania-somnifera.

[162] New Withanolides and Other Constituents from the Fruit of Withania somnifera—Abou-Douh—2002—Archiv Der Pharmazie—Wiley Online Library. https://onlinelibrary.wiley.com/doi/abs/10.1002/1521-4184%28200208%29335%3A6%3C267%3A%3AAID-ARDP267%3E3.0.CO%3B2-E.

[163] Jayaprakasam B., Strasburg G.A., Nair M.G. (2004). Potent Lipid Peroxidation Inhibitors from *Withania somnifera* Fruits. Tetrahedron.

[164] Abutaha N. (2015). In Vitro Antiproliferative Activity of Partially Purified *Withania somnifera* Fruit Extract on Different Cancer Cell Lines. J. BUON Off. J. Balk. Union Oncol..

[165] Ichikawa H., Takada Y., Shishodia S., Jayaprakasam B., Nair M.G., Aggarwal B.B. (2006). Withanolides Potentiate Apoptosis, Inhibit Invasion, and Abolish Osteoclastogenesis through Suppression of Nuclear Factor-ΚB (NF-ΚB) Activation and NF-ΚB–Regulated Gene Expression. Mol. Cancer Ther..

[166] Patel S.B., Rao N.J., Hingorani L.L. (2016). Safety Assessment of *Withania somnifera* Extract Standardized for Withaferin A: Acute and Sub-Acute Toxicity Study. J. Ayurveda Integr. Med..

[167] Prabu P.C., Panchapakesan S., Raj C.D. (2013). Acute and Sub-Acute Oral Toxicity Assessment of the Hydroalcoholic Extract of *Withania somnifera* Roots in Wistar Rats. Phytother. Res..

[168] Sharada A.C., Solomon F.E., Devi P.U. (1993). Toxicity of *Withania somnifera* Root Extract in Rats and Mice. Int. J. Pharmacogn..

[169] Balkrishna A., Sinha S., Srivastava J., Varshney A. (2022). *Withania somnifera* (L.) Dunal Whole-Plant Extract Demonstrates Acceptable Non-Clinical Safety in Rat 28-Day Subacute Toxicity Evaluation under GLP-Compliance. Sci. Rep..

[170] Raut A.A., Rege N.N., Tadvi F.M., Solanki P.V., Kene K.R., Shirolkar S.G., Pandey S.N., Vaidya R.A., Vaidya A.B. (2012). Exploratory Study to Evaluate Tolerability, Safety, and Activity of Ashwagandha (*Withania somnifera*) in Healthy Volunteers. J. Ayurveda Integr. Med..

[171] Tandon N., Yadav S.S. (2020). Safety and Clinical Effectiveness of *Withania somnifera* (Linn.) Dunal Root in Human Ailments. J. Ethnopharmacol..

[172] Xylopia Aethiopica—Useful Tropical Plants. https://tropical.theferns.info/viewtropical.php?id=Xylopia+aethiopica.

[173] Fetse J.P., Kofie W., Adosraku R.K. (2016). Ethnopharmacological Importance of *Xylopia aethiopica* (DUNAL) A. RICH (Annonaceae)—A Review. J. Pharm. Res. Int..

[174] Earnest E. (2014). *Xylopia aethiopica*: A Review of Its Ethnomedicinal, Chemical and Pharmacological Properties. Am. J. Pharmtech Res..

[175] Yin X., Chávez León M.A.S.C., Osae R., Linus L.O., Qi L.-W., Alolga R.N. (2019). *Xylopia aethiopica* Seeds from Two Countries in West Africa Exhibit Differences in Their Proteomes, Mineral Content and Bioactive Phytochemical Composition. Molecules.

[176] The Useful Plants of West Tropical Africa|SpringerLink. https://link.springer.com/article/10.1007/BF02859140.

[177] Abubakar I.B., Ukwuani-Kwaja A.N., Olayiwola F.S., Malami I., Muhammad A., Ahmed S.J., Nurudeen Q.O., Falana M.B. (2020). An Inventory of Medicinal Plants Used for Treatment of Cancer in Kwara and Lagos State, Nigeria. Eur. J. Integr. Med..

[178] Moreira I.C., Roque N.F., Vilegas W., Zalewski C.A., Lago J.H.G., Funasaki M. (2013). Genus Xylopia (Annonaceae): Chemical and Biological Aspects. Chem. Biodivers..

[179] Adaramoye O.A., Sarkar J., Singh N., Meena S., Changkija B., Yadav P.P., Kanojiya S., Sinha S. (2011). Antiproliferative Action of *Xylopia aethiopica* Fruit Extract on Human Cervical Cancer Cells. Phytother. Res..

[180] Choumessi A.T., Loureiro R., Silva A.M., Moreira A.C., Pieme A.C., Tazoacha A., Oliveira P.J., Penlap V.B. (2012). Toxicity Evaluation of Some Traditional African Spices on Breast Cancer Cells and Isolated Rat Hepatic Mitochondria. Food Chem. Toxicol..

[181] Choumessi A.T., Danel M., Chassaing S., Truchet I., Penlap V.B., Pieme A.C., Asonganyi T., Ducommun B., Valette A. (2012). Characterization of the Antiproliferative Activity of *Xylopia aethiopica*. Cell Div..

[182] Kuete V., Krusche B., Youns M., Voukeng I., Fankam A.G., Tankeo S., Lacmata S., Efferth T. (2011). Cytotoxicity of Some Cameroonian Spices and Selected Medicinal Plant Extracts. J. Ethnopharmacol..

[183] Kuete V., Sandjo L.P., Wiench B., Efferth T. (2013). Cytotoxicity and Modes of Action of Four Cameroonian Dietary Spices Ethno-Medically Used to Treat Cancers: *Echinops Giganteus*, *Xylopia aethiopica*, *Imperata Cylindrica* and *Piper Capense*. J. Ethnopharmacol..

[184] Kuete V., Sandjo L.P., Mbaveng A.T., Zeino M., Efferth T. (2015). Cytotoxicity of Compounds from *Xylopia aethiopica* towards Multi-Factorial Drug-Resistant Cancer Cells. Phytomedicine.

[185] Asekun O.T., Adeniyi B.A. (2004). Antimicrobial and Cytotoxic Activities of the Fruit Essential Oil of *Xylopia aethiopica* from Nigeria. Fitoterapia.

[186] Ribeiro V., Ferreres F., Macedo T., Gil-Izquierdo Á., Oliveira A.P., Gomes N.G.M., Araújo L., Pereira D.M., Andrade P.B., Valentão P. (2021). Activation of Caspase-3 in Gastric Adenocarcinoma AGS Cells by *Xylopia aethiopica* (Dunal) A. Rich. Fruit and Characterization of Its Phenolic Fingerprint by HPLC-DAD-ESI(Ion Trap)-MSn and UPLC-ESI-QTOF-MS2. Food Res. Int..

[187] Akinloye O.A., Ayodele P.F., Ore A. (2019). Median Lethality Dose of *Xylopia aethiopica* Fruit Ethanol Extract. J. Anal. Tech. Res..

[188] Assih M., Badjabaïssi E., Bescond J., Mouzou A., Pakoussi T., Sanvee S.C.J., Yérima M., Diallo A., Dossou-Yovo K.M., Kaboua K. (2022). Toxicological Studies of Hydroethanolic Leaf Extract of *Xylopia aethiopica* (Dunal) A. Rich. (Annonaceae) on Wistar Rats. J. Drug Deliv. Ther..

[189] Keren A.O., Sani G.H., Vandi Z.J. (2022). Acute Toxicity Study of Aqueous Fruit Extract of *Xylopia aethiopica* (Dunal) a. Rich. In Albino Rats. Kanem J. Med. Sci..

[190] Physico-Chemical and Toxicological Studies On Xylopia Aethropica Leaves. https://www.researchgate.net/publication/275646964_PHYSICO-CHEMICAL_AND_TOXICOLOGICAL_STUDIES_ON_XYLOPIA_AETHROPICA_LEAVES.

[191] Ibitoye D.O., Kolawole A.O. (2022). Farmers’ Appraisal on Okra [*Abelmoschus esculentus* (L.)] Production and Phenotypic Characterization: A Synergistic Approach for Improvement. Front. Plant Sci..

[192] CABI (2022). Abelmoschus esculentus (Okra).

[193] Elkhalifa A.E.O., Alshammari E., Adnan M., Alcantara J.C., Awadelkareem A.M., Eltoum N.E., Mehmood K., Panda B.P., Ashraf S.A. (2021). Okra (*Abelmoschus esculentus*) as a Potential Dietary Medicine with Nutraceutical Importance for Sustainable Health Applications. Molecules.

[194] Taiwo B.J., Popoola T.D., van Heerden F.R., Fatokun A.A. (2021). Isolation and Characterisation of Two Quercetin Glucosides with Potent Anti-Reactive Oxygen Species (ROS) Activity and an Olean-12-En Triterpene Glucoside from the Fruit of *Abelmoschus esculentus* (L.) Moench. Chem. Biodivers..

[195] Phytochemical Information and Pharmacological Activities of Okra (Abelmoschus esculentus): A Literature-based Review. https://onlinelibrary.wiley.com/doi/epdf/10.1002/ptr.6212.

[196] Agregán R., Pateiro M., Bohrer B.M., Shariati M.A., Nawaz A., Gohari G., Lorenzo J.M. (2022). Biological Activity and Development of Functional Foods Fortified with Okra (*Abelmoschus esculentus*). Crit. Rev. Food Sci. Nutr..

[197] Romdhane M.H., Chahdoura H., Barros L., Dias M.I., Corrêa R.C.G., Morales P., Ciudad-Mulero M., Flamini G., Majdoub H., Ferreira I.C.F.R. (2020). Chemical Composition, Nutritional Value, and Biological Evaluation of Tunisian Okra Pods (*Abelmoschus esculentus* L. Moench). Molecules.

[198] Chaemsawang W., Prasongchean W., Papadopoulos K.I., Ritthidej G., Sukrong S., Wattanaarsakit P. (2019). The Effect of Okra (*Abelmoschus esculentus* (L.) Moench) Seed Extract on Human Cancer Cell Lines Delivered in Its Native Form and Loaded in Polymeric Micelles. Int. J. Biomater..

[199] Ping M.-H. (2020). Hyperin Controls the Development and Therapy of Gastric Cancer via Regulating Wnt/β-Catenin Signaling. Cancer Manag. Res..

[200] Monte L.G., Santi-Gadelha T., Reis L.B., Braganhol E., Prietsch R.F., Dellagostin O.A., e Lacerda R.R., Gadelha C.A.A., Conceição F.R., Pinto L.S. (2014). Lectin of *Abelmoschus esculentus* (Okra) Promotes Selective Antitumor Effects in Human Breast Cancer Cells. Biotechnol. Lett..

[201] Musthafa S.A., Muthu K., Vijayakumar S., George S.J., Murali S., Govindaraj J., Munuswamy-Ramanujam G. (2021). Lectin Isolated from *Abelmoschus esculentus* Induces Caspase Mediated Apoptosis in Human U87 Glioblastoma Cell Lines and Modulates the Expression of Circadian Clock Genes. Toxicon.

[202] Ahmed H.E., Iqbal Y., Aziz M.H., Atif M., Batool Z., Hanif A., Yaqub N., Farooq W.A., Ahmad S., Fatehmulla A. (2021). Green Synthesis of CeO_2_ Nanoparticles from the *Abelmoschus esculentus* Extract: Evaluation of Antioxidant, Anticancer, Antibacterial, and Wound-Healing Activities. Molecules.

[203] Safety Evaluation of Abelmoschus Esculentus Polysaccharide|Request PDF. https://www.researchgate.net/publication/288097801_Safety_evaluation_of_Abelmoschus_esculentus_polysaccharide.

[204] Doreddula S.K., Bonam S.R., Gaddam D.P., Desu B.S.R., Ramarao N., Pandy V. (2014). Phytochemical Analysis, Antioxidant, Antistress, and Nootropic Activities of Aqueous and Methanolic Seed Extracts of Ladies Finger (*Abelmoschus esculentus* L.) in Mice. Sci. World J..

[205] Sabitha V., Ramachandran S., Naveen K.R., Panneerselvam K. (2012). Investigation of in Vivo Antioxidant Property of *Abelmoschus esculentus* (L) Moench. Fruit Seed and Peel Powders in Streptozotocin-Induced Diabetic Rats. J. Ayurveda Integr. Med..

[206] Esmaeilzadeh D., Razavi B.M., Hosseinzadeh H. (2020). Effect of *Abelmoschus esculentus* (Okra) on Metabolic Syndrome: A Review. Phytother. Res..

[207] Taniguchi-Fukatsu A., Yamanaka-Okumura H., Naniwa-Kuroki Y., Nishida Y., Yamamoto H., Taketani Y., Takeda E. (2012). Natto and Viscous Vegetables in a Japanese-Style Breakfast Improved Insulin Sensitivity, Lipid Metabolism and Oxidative Stress in Overweight Subjects with Impaired Glucose Tolerance. Br. J. Nutr..

[208] Jenkins D.J.A., Kendall C.W.C., Marchie A., Faulkner D.A., Wong J.M.W., de Souza R., Emam A., Parker T.L., Vidgen E., Trautwein E.A. (2005). Direct Comparison of a Dietary Portfolio of Cholesterol-Lowering Foods with a Statin in Hypercholesterolemic Participants. Am. J. Clin. Nutr..

[209] Moradi A., Tarrahi M.-J., Ghasempour S., Shafiepour M., Clark C.C.T., Safavi S.-M. (2020). The Effect of Okra (*Abelmoschus esculentus*) on Lipid Profiles and Glycemic Indices in Type 2 Diabetic Adults: Randomized Double Blinded Trials. Phytother. Res..

[210] CABI (2019). Carissa macrocarpa (Natal Plum).

[211] Orabi M.A.A., Khalil H.M.A., Abouelela M.E., Zaafar D., Ahmed Y.H., Naggar R.A., Alyami H.S., Abdel-Sattar E.-S., Matsunami K., Hamdan D.I. (2021). *Carissa macrocarpa* Leaves Polar Fraction Ameliorates Doxorubicin-Induced Neurotoxicity in Rats via Downregulating the Oxidative Stress and Inflammatory Markers. Pharmaceuticals.

[212] Castañeda-Loaiza V., Placines C., Rodrigues M.J., Pereira C., Zengin G., Uysal A., Jeko J., Cziáky Z., Reis C.P., Gaspar M.M. (2020). If You Cannot Beat Them, Join Them: Exploring the Fruits of the Invasive Species *Carpobrotus edulis* (L.) N.E. Br as a Source of Bioactive Products. Ind. Crops Prod..

[213] Mudimba T.N., Nguta J.M. (2019). Traditional Uses, Phytochemistry and Pharmacological Activity of *Carpobrotus edulis*: A Global Perspective. J. Phytopharm..

[214] Akhalwaya S., van Vuuren S., Patel M. (2018). An in Vitro Investigation of Indigenous South African Medicinal Plants Used to Treat Oral Infections. J. Ethnopharmacol..

[215] Cock I.E., van Vuuren S.F. (2014). Anti-Proteus Activity of Some South African Medicinal Plants: Their Potential for the Prevention of Rheumatoid Arthritis. Inflammopharmacology.

[216] (PDF) Acute and Subacute Toxicity Evaluation of Aqueous Extracts of Carpobrotus edulis in Sprague Dawley Rats. https://www.researchgate.net/publication/343681218_Acute_and_subacute_toxicity_evaluation_of_aqueous_extracts_of_Carpobrotus_edulis_in_Sprague_Dawley_rats?_iepl%5BgeneralViewId%5D=BlZqYO6VIoqAmmtEkUdiIIV289LMJZ1wBNXD&_iepl%5Bcontexts%5D%5B0%5D=searchReact&_iepl%5BviewId%5D=ZQY1klgoMLqybb34NSG0QsBibNPeY1XMFwcU&_iepl%5BsearchType%5D=publication&_iepl%5Bdata%5D%5BcountLessEqual20%5D=1&_iepl%5Bdata%5D%5BinteractedWithPosition1%5D=1&_iepl%5Bdata%5D%5BwithoutEnrichment%5D=1&_iepl%5Bposition%5D=1&_iepl%5BrgKey%5D=PB%3A343681218&_iepl%5BtargetEntityId%5D=PB%3A343681218&_iepl%5BinteractionType%5D=publicationTitle.

[217] Ayyanar M., Subash-Babu P. (2012). *Syzygium cumini* (L.) Skeels: A Review of Its Phytochemical Constituents and Traditional Uses. Asian Pac. J. Trop. Biomed..

[218] Chagas V.T., França L.M., Malik S., Paes A.M. (2015). *Syzygium cumini* (L.) Skeels: A Prominent Source of Bioactive Molecules against Cardiometabolic Diseases. Front. Pharmacol..

[219] Aqil F., Gupta A., Munagala R., Jeyabalan J., Kausar H., Sharma R.J., Singh I.P., Gupta R.C. (2012). Antioxidant and Antiproliferative Activities of Anthocyanin/Ellagitannin-Enriched Extracts From *Syzygium cumini* L. (Jamun, the Indian Blackberry). Nutr. Cancer.

[220] The Anthocyanin Components and Cytotoxic Activity of Syzygium cumini (L.) Fruits Growing in Egypt -Natural Product Sciences|Korea Science. https://koreascience.kr/article/JAKO200724737574177.page.

[221] Li L., Adams L.S., Chen S., Killian C., Ahmed A., Seeram N.P. (2009). Eugenia Jambolana Lam. Berry Extract Inhibits Growth and Induces Apoptosis of Human Breast Cancer but Not Non-Tumorigenic Breast Cells. J. Agric. Food Chem..

[222] Barh D., Viswanathan G. Syzygium cumini Inhibits Growth and Induces Apoptosis in Cervical Cancer Cell Lines: A Primary Study. http://ecancer.org/en/journal/article/83-syzygium-cumini-inhibits-growth-and-induces-apoptosis-in-cervical-cancer-cell-lines-a-primary-study.

[223] Parmar J., Sharma P., Verma P., Goyal P.K. (2010). Chemopreventive Action of *Syzygium cumini* on DMBA-Induced Skin Papillomagenesis in Mice. Asian Pac. J. Cancer Prev..

[224] (2010). Evaluation of Anti-Cancer and Anti-Oxidative Potential of *Syzygium cumini* Against Benzo[a]Pyrene (BaP) Induced Gastric Carcinogenesis in Mice. Asian Pac. J. Cancer Prev..

[225] Kumar M., Zhang B., Nishad J., Verma A., Sheri V., Dhumal S., Radha, Sharma N., Chandran D., Senapathy M. (2022). Jamun (*Syzygium cumini* (L.) Skeels) Seed: A Review on Nutritional Profile, Functional Food Properties, Health-Promoting Applications, and Safety Aspects. Processes.

[226] Chaturvedi A., Kumar M.M., Bhawani G., Chaturvedi H., Kumar M., Goel R.K. (2007). Effect of Ethanolic Extract of Eugenia Jambolana Seeds on Gastric Ulceration and Secretion in Rats. Indian J. Physiol. Pharmacol..

[227] Kumar E.K., Mastan S.K., Reddy K.R., Reddy G.A., Raghunandan N., Chaitanya G. (2009). Antiarthrtic Property of Methanolic Extract of *Syzygium cumini* Seeds. Int. J. Integr. Biol..

[228] Silva S.D.N., Abreu I.C., Silva G.F.C., Ribeiro R.M., Lopes A.D.S., Cartágenes M.D.S.D.S., Freire S.M.D.F., Borges A.C.R., Borges M.O.D.R. (2012). The Toxicity Evaluation of *Syzygium cumini* Leaves in Rodents. Rev. Bras. Farmacogn..

[229] Qamar M., Akhtar S., Ismail T., Wahid M., Ali S., Nazir Y., Murtaza S., Abbas M.W., Ziora Z.M. (2022). *Syzygium cumini* (L.) Skeels Extracts; in Vivo Anti-Nociceptive, Anti-Inflammatory, Acute and Subacute Toxicity Assessment. J. Ethnopharmacol..

[230] Teixeira C.C., Fuchs F.D., Weinert L.S., Esteves J. (2006). The Efficacy of Folk Medicines in the Management of Type 2 Diabetes Mellitus: Results of a Randomized Controlled Trial of *Syzygium cumini* (L.) Skeels. J. Clin. Pharm. Ther..

[231] Teixeira C.C., Rava C.A., Mallman da Silva P., Melchior R., Argenta R., Anselmi F., Almeida C.R.C., Fuchs F.D. (2000). Absence of Antihyperglycemic Effect of Jambolan in Experimental and Clinical Models. J. Ethnopharmacol..

[232] Shivaprakash G., Pai M.R.S.M., Mangalore N., Kumarchandra R., Acharya S., Kuppusamy R., Shirwaikar A., Adhikari M.R., Ganesh J. (2011). Antioxidant Potential of Eugenia Jambolana Seed; A Randomized Clinical Trial in Type 2 Diabetes Mellitus. Int. J. Pharma Bio Sci..

[233] Acharya S., Shivaprakash G., Baliga R., Adhikari P., Jyothi G., Pai M.R.S.M. (2010). Effect of Eugenia Jambolana on Plasma Glucose, Insulin Sensitivity and HDL-C Levels: Preliminary Results of a Randomized Clinical Trial. J. Pharm. Res..

[234] Kigelia Africana|PlantZAfrica. https://pza.sanbi.org/kigelia-africana.

[235] Bello I., Shehu M.W., Musa M., Asmawi M.Z., Mahmud R. (2016). *Kigelia africana* (Lam.) Benth. (Sausage Tree): Phytochemistry and Pharmacological Review of a Quintessential African Traditional Medicinal Plant. J. Ethnopharmacol..

[236] Ramadan K.M.A., El-Beltagi H.S., Mohamed H.I., Shalaby T.A., Galal A., Mansour A.T., Aboul Fotouh M.M., Bendary E.S.A. (2022). Antioxidant, Anti-Cancer Activity and Phytochemicals Profiling of *Kigelia pinnata* Fruits. Separations.

[237] Osman A.G., Ali Z., Chittiboyina A.G., Khan I.A. (2017). *Kigelia africana* Fruit: Constituents, Bioactivity, and Reflection on Composition Disparities. World J. Tradit. Chin. Med..

[238] Gouda Y.G., Abdel-baky A.M., Darwish F.M., Mohamed K.M., Kasai R., Yamasaki K. (2003). Iridoids from *Kigelia pinnata* DC. Fruits. Phytochemistry.

[239] Houghton P., Jäger A.K. (2002). The Sausage Tree (*Kigelia pinnata*): Ethnobotany and Recent Scientific Work. S. Afr. J. Bot..

[240] Jackson S.J., Houghton P.J., Retsas S., Photiou A. (2000). In Vitro Cytotoxicity of Norviburtinal and Isopinnatal from *Kigelia pinnata* Against Cancer Cell Lines. Planta Med..

[241] Higgins C.A., Bell T., Delbederi Z., Feutren-Burton S., McClean B., O’Dowd C., Watters W., Armstrong P., Waugh D., Berg H. (2010). van den Growth Inhibitory Activity of Extracted Material and Isolated Compounds from the Fruits of *Kigelia pinnata*. Planta Med..

[242] Chivandi E., Cave E., Davidson B.C., Erlwanger K.H., Moyo D., Madziva M.T. (2012). Suppression of Caco-2 and HEK-293 Cell Proliferation by *Kigelia africana*, *Mimusops zeyheri* and Ximenia Caffra Seed Oils. In Vivo.

[243] Azuine M.A., Ibrahim K., Enwerem N.M., Wambebe C., Kolodziej H. (1997). Protective Role of *Kigelia africana* Fruits against Benzo[a]Pyrene-Induced Forestomach Tumourigenesis in Mice and against Albumen-Induced Inflammation in Rats. Pharm. Pharmacol. Lett..

[244] Olufemi A., Omotayo O., David A., Monjeed I., Adebola O., Bilikis S., Temitope A. (2017). *Kigelia africana* Stem Bark, Fruit and Leaf Extracts Alleviate Benzene-Induced Leukaemia in Rats. J. Pharm. Res. Int..

[245] Plants Traditionally Used Individually and in Combination to Treat Sexually Transmitted Infections in Northern Maputaland, South Africa: Antimicrobial Activity and Cytotoxicity—ScienceDirect. https://www.sciencedirect.com/science/article/pii/S0378874113005072.

[246] Khan M.A.A., Islam M.T. (2012). Analgesic and Cytotoxic Activity of *Acorus calamus* L., *Kigelia pinnata* L., *Mangifera indica* L. and *Tabernaemontana divaricata* L.. J. Pharm. Bioallied Sci..

[247] Hassan S.W., Mshelia P.Y., Abubakar M.G., Adamu Y.A., Yakubu A.S. (2015). Wound Healing, Antioxidants and Toxicological Properties of Root Extracts of *Kigelia africana* (Lam.) Benth. Int. J. Sci. Basic Appl. Res. IJSBAR.

[248] Kh H.K. (2023). Evaluation of anti-ulcer activity of methanolic extracts of *Kigelia africana*, *Sophora interrupta* and *Holoptelea integrifolia* leaves in experimental rats. Int. J. Curr. Pharm. Res..

[249] Fredrick A., Ebele O., Obi P. (2014). Analgesic, Phytochemical and Toxicological Investigations Of Ethanol Extract Of The Leaves Of *Kigelia africana* (Lam.) Benth (Family Bignoniaceae)-Sausage Tree. J. Pharm. Biomed. Sci..

[250] Akah P.A. (1996). Antidiarrheal Activity of *Kigelia africana* in Experimental Animals. J. Herbs Spices Med. Plants.

[251] Analgesic and Anti-Inflammatory Activities of Ethanolic Extract of the Stem Bark of Kigelia africana in Wistar Albino Mice and Rats. https://www.researchgate.net/publication/284182893_Analgesic_and_anti-inflammatory_activities_of_ethanolic_extract_of_the_stem_bark_of_Kigelia_africana_in_Wistar_albino_mice_and_rats.

[252] Abdul Wahab S.M., Jantan I., Haque M.A., Arshad L. (2018). Exploring the Leaves of *Annona muricata* L. as a Source of Potential Anti-Inflammatory and Anticancer Agents. Front. Pharmacol..

[253] Anticancer Properties of Graviola (Annona muricata): A Comprehensive Mechanistic Review—PMC. https://www.ncbi.nlm.nih.gov/pmc/articles/PMC6091294/.

[254] Jaramillo M.C., Arango G.J., González M.C., Robledo S.M., Velez I.D. (2000). Cytotoxicity and Antileishmanial Activity of *Annona muricata* Pericarp. Fitoterapia.

[255] Sun S., Liu J., Zhou N., Zhu W., Dou Q.P., Zhou K. (2016). Isolation of Three New Annonaceous Acetogenins from Graviola Fruit (*Annona muricata*) and Their Anti-Proliferation on Human Prostate Cancer Cell PC-3. Bioorg. Med. Chem. Lett..

[256] Liaw C.-C., Chang F.-R., Lin C.-Y., Chou C.-J., Chiu H.-F., Wu M.-J., Wu Y.-C. (2002). New Cytotoxic Monotetrahydrofuran Annonaceous Acetogenins from *Annona muricata*. J. Nat. Prod..

[257] Dai Y., Hogan S., Schmelz E.M., Ju Y.H., Canning C., Zhou K. (2011). Selective Growth Inhibition of Human Breast Cancer Cells by Graviola Fruit Extract In Vitro and In Vivo Involving Downregulation of EGFR Expression. Nutr. Cancer.

[258] Mutakin M., Fauziati R., Fadhilah F.N., Zuhrotun A., Amalia R., Hadisaputri Y.E. (2022). Pharmacological Activities of Soursop (*Annona muricata* Lin.). Molecules.

[259] De Sousa O.V., Vieira G.D.-V., De Pinho J.d.J.R.G., Yamamoto C.H., Alves M.S. (2010). Antinociceptive and Anti-Inflammatory Activities of the Ethanol Extract of *Annona muricata* L. Leaves in Animal Models. Int. J. Mol. Sci..

[260] Arthur F.K.N., Woode E., Terlabi E.O., Larbie C. (2011). Evaluation of Acute and Subchronic Toxicity of *Annona muricata* (Linn.) Aqueous Extract in Animals. Eur. J. Exp. Biol..

[261] Boyom F.F., Fokou P.V.T., Yamthe L.R.T., Mfopa A.N., Kemgne E.M., Mbacham W.F., Tsamo E., Zollo P.H.A., Gut J., Rosenthal P.J. (2011). Potent Antiplasmodial Extracts from Cameroonian Annonaceae. J. Ethnopharmacol..

[262] Quilez A.M., Montserrat-de la Paz S., De La Puerta R., Fernández-Arche M.A., Gargia-Gimenez M.D. (2015). Validation of Ethnopharmacological Use as Anti-Inflammatory of a Decoction from *Annona muricata* Leaves. Afr. J. Tradit. Complement. Altern. Med..

[263] Yasir M., Das S., Kharya M.D. (2010). The Phytochemical and Pharmacological Profile of *Persea americana* Mill. Pharmacogn. Rev..

[264] The Avocado (Persea americana, Lauraceae) Crop in Mesoamerica: 10,000 Years of History on JSTOR. https://www.jstor.org/stable/41761865.

[265] Persea Americana—Useful Tropical Plants. https://tropical.theferns.info/viewtropical.php?id=Persea+americana.

[266] Ashande C. (2019). A Mini-Review on the Phytochemistry and Pharmacology of the Medicinal Plant Species *Persea americana* Mill. (Lauraceae). J. Nat. Prod. Res. Ethnopharmacol..

[267] Bhuyan D.J., Alsherbiny M.A., Perera S., Low M., Basu A., Devi O.A., Barooah M.S., Li C.G., Papoutsis K. (2019). The Odyssey of Bioactive Compounds in Avocado (*Persea americana*) and Their Health Benefits. Antioxidants.

[268] Jackson M.D., Walker S.P., Simpson-Smith C.M., Lindsay C.M., Smith G., McFarlane-Anderson N., Bennett F.I., Coard K.C.M., Aiken W.D., Tulloch T. (2012). Associations of Whole-Blood Fatty Acids and Dietary Intakes with Prostate Cancer in Jamaica. Cancer Causes Control.

[269] Gouegni E.F., Abubakar H. (2013). Phytochemical, Toxicological, Biochemical and Haematological Studies on Avocado (*Persea americana*) in Experimental Animals. Niger. Food J..

[270] Ozolua R., Anaka O., Okpo S., Idogun S. (2009). Acute and Sub-Acute Toxicological Assessment of the Aqueous Seed Extract of *Persea americana* Mill (Lauraceae) in Rats. Afr. J. Tradit. Complement. Altern. Med..

[271] Iheagwam F.N., Onyido B.C., Chinedu S.N. (2023). Toxicological Profile of *Dacryodes edulis* (G. Don.) H. J. Lam. and *Persea americana* M. Seeds in Male Wistar Rats. Comp. Clin. Pathol..

[272] Pahua-Ramos M.E., Ortiz-Moreno A., Chamorro-Cevallos G., Hernández-Navarro M.D., Garduño-Siciliano L., Necoechea-Mondragón H., Hernández-Ortega M. (2012). Hypolipidemic Effect of Avocado (*Persea americana* Mill) Seed in a Hypercholesterolemic Mouse Model. Plant Foods Hum. Nutr..

[273] Orabueze I.C., Babalola R., Azuonwu O., Okoko I.-I., Asare G. (2021). Evaluation of Possible Effects of *Persea americana* Seeds on Female Reproductive Hormonal and Toxicity Profile. J. Ethnopharmacol..

[274] Rao U.S.M., Adinew B. (2011). Remnant B-Cell-Stimulative and Anti-Oxidative Effects of *Persea americana* Fruit Extract Studied in Rats Introduced into Streptozotocin—Induced Hyperglycaemic State. Afr. J. Tradit. Complement. Altern. Med..

[275] Nicolella H.D., Neto F.R., Corrêa M.B., Lopes D.H., Rondon E.N., dos Santos L.F.R., de Oliveira P.F., Damasceno J.L., Acésio N.O., Turatti I.C.C. (2017). Toxicogenetic Study of *Persea americana* Fruit Pulp Oil and Its Effect on Genomic Instability. Food Chem. Toxicol..

[276] Paul R., Kulkarni P., Ganesh N. (2011). Avocado Fruit (*Persea americana* Mill) Exhibits Chemo-Protective Potentiality against Cyclophosphamide Induced Genotoxicity in Human Lymphocyte Culture. J. Exp. Ther. Oncol..

[277] Kulkarni P., Paul R., Ganesh N. (2010). In Vitro Evaluation of Genotoxicity of Avocado (*Persea americana*) Fruit and Leaf Extracts in Human Peripheral Lymphocytes. J. Environ. Sci. Health Part C Environ. Carcinog. Ecotoxicol. Rev..

[278] Oelrichs P.B., Ng J.C., Seawright A.A., Ward A., Schäffeler L., Macleod J.K. (1995). Isolation and Identification of a Compound from Avocado (*Persea americana*) Leaves Which Causes Necrosis of the Acinar Epithelium of the Lactating Mammary Gland and the Myocardium. Nat. Toxins.

[279] Freitas M.S., Pereira A.H.B., Pereira G.O., Menezes I.S., Lucena A.R., Almeida C.R.F., Pereira E.G., Santos L.A., Tozin L.R.S., Alves F.M. (2022). Acetogenin-Induced Fibrotic Heart Disease from Avocado (*Persea americana*, Lauraceae) Poisoning in Horses. Toxicon.

[280] Craigmill A., Seawright A., Mattila T., Frost A. (1989). Pathological Changes in the Mammary Gland and Biochemical Changes in Milk of the Goat Following Oral Dosing with Leaf of the Avocado (*Persea americana*). Aust. Vet. J..

[281] Grant R., Basson P.A., Booker H.H., Hofherr J.B., Anthonissen M. (1991). Cardiomyopathy Caused by Avocado (*Persea americana* Mill) Leaves. J. S. Afr. Vet. Assoc..

[282] Buoro I.B., Nyamwange S.B., Chai D., Munyua S.M. (1994). Putative Avocado Toxicity in Two Dogs. Onderstepoort J. Vet. Res..

[283] Aguirre L.S., Sandoval G.V., Medina D.M., Martinez O.G., Micheloud J.F. (2019). Acute Heart Failure in Rabbits by Avocado Leaf Poisoning. Toxicon Off. J. Int. Soc. Toxinology.

[284] Burger W.P., Naudé T.W., Van Rensburg I.B., Botha C.J., Pienaar A.C. (1994). Cardiomyopathy in Ostriches (*Struthio camelus*) Due to Avocado (*Persea americana* Var. Guatemalensis) Intoxication. J. S. Afr. Vet. Assoc..

[285] Maphetu N., Unuofin J.O., Masuku N.P., Olisah C., Lebelo S.L. (2022). Medicinal Uses, Pharmacological Activities, Phytochemistry, and the Molecular Mechanisms of *Punica granatum* L. (Pomegranate) Plant Extracts: A Review. Biomed. Pharmacother..

[286] Rahimi H.R., Arastoo M., Ostad S.N. (2012). A Comprehensive Review of *Punica granatum* (Pomegranate) Properties in Toxicological, Pharmacological, Cellular and Molecular Biology Researches. Iran. J. Pharm. Res. IJPR.

[287] Medicinal Uses of Punica granatum and Its Health Benefits. https://www.phytojournal.com/vol1Issue5/6.html.

[288] Fakudze N.T., Aniogo E.C., George B.P., Abrahamse H. (2022). The Therapeutic Efficacy of *Punica granatum* and Its Bioactive Constituents with Special Reference to Photodynamic Therapy. Plants.

[289] Panth N., Manandhar B., Paudel K.R. (2017). Anticancer Activity of *Punica granatum* (Pomegranate): A Review. Phytother. Res..

[290] Zarfeshany A., Asgary S., Javanmard S.H. (2014). Potent Health Effects of Pomegranate. Adv. Biomed. Res..

[291] Freedland S.J., Carducci M., Kroeger N., Partin A., Rao J., Jin Y., Kerkoutian S., Wu H., Li Y., Creel P. (2013). A Double-Blind, Randomized, Neoadjuvant Study of the Tissue Effects of POMx Pills in Men with Prostate Cancer Before Radical Prostatectomy. Cancer Prev. Res..

[292] Pantuck A.J., Leppert J.T., Zomorodian N., Aronson W., Hong J., Barnard R.J., Seeram N., Liker H., Wang H., Elashoff R. (2006). Phase II Study of Pomegranate Juice for Men with Rising Prostate-Specific Antigen Following Surgery or Radiation for Prostate Cancer. Clin. Cancer Res..

[293] Paller C.J., Ye X., Wozniak P.J., Gillespie B.K., Sieber P.R., Greengold R.H., Stockton B.R., Hertzman B.L., Efros M.D., Roper R.P. (2013). A Randomized Phase II Study of Pomegranate Extract for Men with Rising PSA Following Initial Therapy for Localized Prostate Cancer. Prostate Cancer Prostatic Dis..

[294] González-Sarrías A., Giménez-Bastida J.A., García-Conesa M.T., Gómez-Sánchez M.B., García-Talavera N.V., Gil-Izquierdo A., Sánchez-Álvarez C., Fontana-Compiano L.O., Morga-Egea J.P., Pastor-Quirante F.A. (2010). Occurrence of Urolithins, Gut Microbiota Ellagic Acid Metabolites and Proliferation Markers Expression Response in the Human Prostate Gland upon Consumption of Walnuts and Pomegranate Juice. Mol. Nutr. Food Res..

[295] Setiadhi R., Sufiawati I., Zakiawati D., Nuraeny N., Hidayat W., Firman D. (2017). Evaluation of Antibacterial Activity and Acute Toxicity of Pomegranate (*Punica granatum* l.) Seed Ethanolic Extracts in Swiss Webster Mice. J. Dentomaxillofacial Sci..

[296] Vidal A., Fallarero A., Peña B.R., Medina M.E., Gra B., Rivera F., Gutierrez Y., Vuorela P.M. (2003). Studies on the Toxicity of *Punica granatum* L. (Punicaceae) Whole Fruit Extracts. J. Ethnopharmacol..

[297] Bassiri-Jahromi S., Pourshafie M., Mirabzadeh E., Tavasoli A., Katiraee F., Mostafavi E., Abbasian S. (2015). *Punica granatum* Peel Extract Toxicity in Mice. Jundishapur J. Nat. Pharm. Prod..

[298] Patel C., Dadhaniya P., Hingorani L., Soni M.G. (2008). Safety Assessment of Pomegranate Fruit Extract: Acute and Subchronic Toxicity Studies. Food Chem. Toxicol. Int. J. Publ. Br. Ind. Biol. Res. Assoc..

[299] Vale E.P., do Rego L.R., Pureza D.D.N., de Barros Silva P.G., de Sousa F.F.O., Neto M.D.A.B.M. (2020). Cytogenetic and Toxicological Effects of *Punica granatum* Linnaeus Fruit Peel Hydroethanolic Extract in Mice. S. Afr. J. Bot..

[300] SciELO—Brazil—Assessment of Mutagenic and Antimutagenic Effects of Punica granatum in Mice Assessment of Mutagenic and Antimutagenic Effects of Punica granatum in Mice. https://www.scielo.br/j/bjps/a/Dbz4zXhPhCN4sK8FqQgy6dw/.

[301] De Amorim A., Borba H.R., Armada J.L. (1995). Test of Mutagenesis in Mice Treated with Aqueous Extracts from *Punica granatum* L.(Pomegranate). Rev. Bras. Farm..

[302] Read E., Deseo M., Hawes M., Rochfort S. (2019). Identification of Potentially Cytotoxic Phenolics Present in Pomegranates (*Punica granatum* L.). Anim. Feed Sci. Technol..

[303] Eghbali S., Askari S.F., Avan R., Sahebkar A. (2021). Therapeutic Effects of *Punica granatum* (Pomegranate): An Updated Review of Clinical Trials. J. Nutr. Metab..

[304] Ntalli N.G., Cottiglia F., Bueno C.A., Alché L.E., Leonti M., Vargiu S., Bifulco E., Menkissoglu-Spiroudi U., Caboni P. (2010). Cytotoxic Tirucallane Triterpenoids from Melia Azedarach Fruits. Molecules.

[305] Adegoke A.M., Gota V., Gupta S., Gbadegesin M.A., Odunola1 O.A. (2021). Evaluation of Antioxidant and Anticancer Activities of Aqueous Extract of the Fruit Pulp of Adansonia Digitata Linn and Its Fractions. Afr. J. Med. Med. Sci..

[306] Ochwang’i D.O., Kimwele C.N., Oduma J.A., Gathumbi P.K., Mbaria J.M., Kiama S.G. (2014). Medicinal Plants Used in Treatment and Management of Cancer in Kakamega County, Kenya. J. Ethnopharmacol..

